# A generalized Knudsen theory for gas transport in disordered porous materials

**DOI:** 10.1038/s41467-026-75656-8

**Published:** 2026-07-16

**Authors:** Jianhao Qian, Ruoyu Wang, Menachem Elimelech

**Affiliations:** 1 Department of Civil and Environmental Engineering, Rice University, Houston, TX USA; 2 Department of Chemical and Biomolecular Engineering, Rice University, Houston, TX USA; 3 Rice Center for Membrane Excellence (RiCeME), Rice University, Houston, TX USA

**Keywords:** Engineering, Nanoscience and technology

## Abstract

Gas transport through nanoporous materials is central to membrane separations, catalysis, and energy technologies. Predicting permeability in these materials is crucial for performance evaluation and material design, but their complex porous network poses significant challenges. Here, we develop a generalized theoretical framework for Knudsen flow in random porous materials. We further derive a concise permeability equation dependent solely on two measurable structural parameters: mean pore size and porosity. Monte Carlo simulations across 5000 random porous networks with porosities ranging from 0 to 0.8 validate the theory with *R*^2^ = 0.985. Non-equilibrium molecular dynamics simulations confirm the applicability of the theory to porous membrane materials, including polymers of intrinsic microporosity, polyamide, and zeolitic imidazolate frameworks. By accounting for molecular size effects, we extend the framework to predict gas selectivity in materials with sub-nanometer pores, showing good agreement with experimental data for weakly adsorbing gases. This work extends Knudsen theory to random porous networks and molecular-sized pores, providing a practical and accessible tool for predicting gas permeability and selectivity in nanoporous materials.

## Abstract (web summary)

Here authors present a theory to predict gas flow in disordered porous materials. Using only porosity and pore size, the model accurately calculates gas permeability and selectivity, which can guide the design of gas separation membranes.

## Introduction

Gas transport through nanoporous networks is fundamental to many gas-involved technologies, including membrane separations^1^, hydrogen storage^2^, catalysis^3^, shale gas extraction^4^, and lithium–air batteries^5^. Due to nanoconfinement, gas molecules collide far more frequently with pore walls than with each other at normal pressures, rendering gas–gas interactions negligible and invalidating classical continuum descriptions such as Darcy’s law^6^. Depending on gas–wall interactions, distinct transport mechanisms emerge^7^, among which Knudsen diffusion dominates when gas–wall interactions are relatively weak, such as for light gases (He, H_2_, N_2_)^8^, water vapor in hydrophobic materials^9^, and gases with high kinetic energy^10^.

Knudsen theory^11^, first formulated by Martin Knudsen in 1909, describes rarefied gas transport through long cylindrical capillaries. In such systems, intermolecular interactions are negligible^12^, making Knudsen theory a natural framework for nanoconfined gas flow. Knudsen theory assumes that gas molecules move ballistically between collisions with the wall and reflect diffusely in random directions according to Lambert’s cosine law^11^. Due to diffuse reflection, molecules entering a channel may reflect back, with only a fraction passing through. Clausing quantified this fraction through the concept of transmission probability^13^, which depends solely on the channel geometry and surface roughness. The gas flux through the channel can then be obtained by multiplying this probability with the entering flux from the feed side, which can be readily calculated using kinetic theory^12^. Subsequent work established analytical expressions for transmission probability for channels with different cross-sections and aspect ratios^12,14^. This framework was further extended to channels with varying surface roughness^15^, particularly atomically smooth channels, where experimentally measured gas permeation rates were found to be several orders of magnitude higher than theoretical predictions under the diffuse reflection assumption^8,16^. Knudsen theory for well-defined channels is now mature, having been extensively validated and widely applied for predicting gas permeability^17–19^.

Real nanoporous structures, however, including porous polymers, metal-organic frameworks, and nanofibrous membranes, as well as nanoscale cracks created by hydraulic fracturing, exhibit complex porous networks^20^. Classical Knudsen theory for simple channel geometries cannot be directly applied to such systems. Several approaches have been proposed to extend Knudsen theory to complex porous networks. A straightforward approach is to approximate the porous network as an assembly of well-defined channels, but this introduces parameters like tortuosity, connectivity, and fractal dimensions that are difficult to obtain^21^. Some frameworks extend models for continuum flow, such as Darcy’s law, by incorporating correction factors to capture Knudsen flow^22^. Yet, characterizing porous Knudsen flow using these methods remains challenging, as both the continuum-based permeability and the specific correction factors are difficult to determine for complex porous networks. Alternatively, numerical methods like the Lattice Boltzmann Method (LBM) and Direct Simulation Monte Carlo (DSMC) can model gas transport more explicitly^23^. Such approaches, however, demand significant computational resources, specialized expertise, and accurate 3D pore structures, which are often difficult to obtain, particularly for complex porous materials.

In this work, we develop a generalized theoretical framework for Knudsen gas flow in random porous structures. By simplifying random porous networks to equivalent straight channels with internal barriers, a simple permeability equation is rigorously derived, depending only on two measurable parameters: porosity and mean pore size (or mean chord length). This theory is validated through Monte Carlo (MC) simulations of randomly generated porous networks with porosities ranging from 0 to 0.8. The framework is further tested for gas transport through porous membrane materials with weak gas-surface interactions. Non-equilibrium molecular dynamics simulations (NEMD) are performed to calculate gas permeability, showing good agreement with theoretical predictions. Additionally, by incorporating molecular size effects, we extend the framework to capture gas selectivity in materials with sub-nanometer pores, obtaining good agreement with experimental data for weakly adsorbing gases.

## Results

### Deriving a general permeability law for random porous networks

**Fig. 1:**
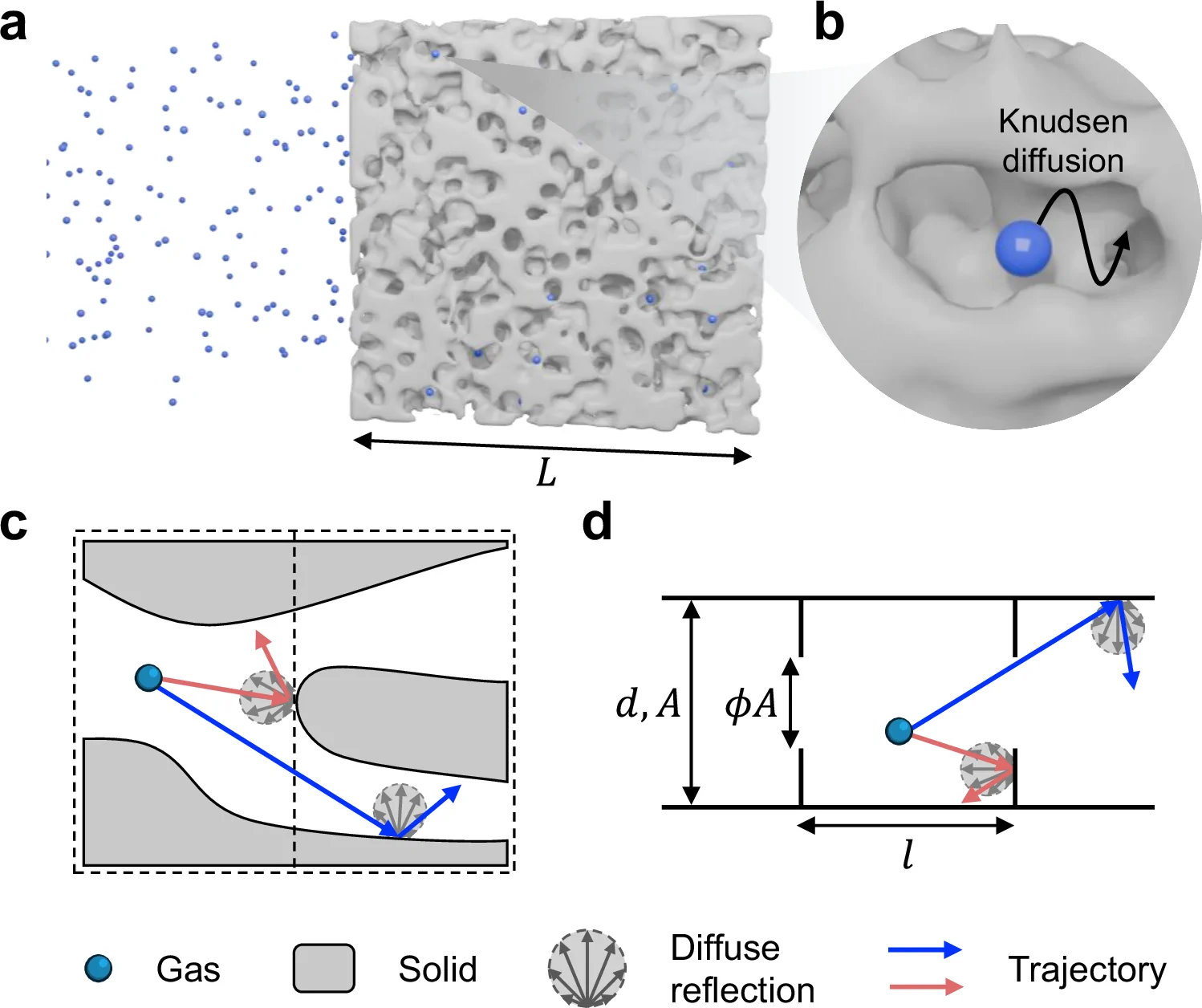
Simplified representation of gas transport in porous networks. **a** Schematic diagram of gas molecules permeating through a random porous material. **b** Illustration of a gas molecule undergoing Knudsen diffusion inside the local pore structure of the porous material. **c** Visualization of the barrier effect during gas transport in random porous networks. Pore walls oriented non-parallel to the flow direction act as geometric obstacles, greatly impeding molecular trajectories and reducing the net gas flux. **d** The equivalent channel model incorporating internal barriers designed to simulate the back-scattering mechanism in random networks. The channel diameter, cross-sectional area, and barrier opening area are denoted as *d*, *A*, and *ϕA*, respectively.

Gas transport through nanoporous networks without well-defined pores is a complex process (Fig. 1a). Local pores differ in shape, connect to varying numbers of neighbors, and partially overlap with adjacent pores (Fig. 1b). For Knudsen diffusion through a well-defined channel, such as a cylindrical pore, the transmission probability or permeability can be derived analytically^24^. Given a known pressure gradient, the local gas pressure above each wall surface element determines the collision rate of gas molecules with that surface element. After reflection from the surface, a calculable fraction of these molecules will pass through the channel outlet cross-section without a second collision. Integrating the flux contributions from all surface elements gives the total gas flux through the channel. For irregular pore networks, however, deriving an analytical expression for gas permeability by summing all wall-element contributions is infeasible.

Simplifying complex porous networks into equivalent, well-defined channel geometries is therefore critical. Conventional approaches typically reduce these networks to straight cylindrical channels, enabling the use of established analytical expressions for Knudsen flux^12^. However, such straight channel models fail to fully account for transport resistance in realistic porous media. In straight channels, resistance arises primarily from diffuse reflections, where gas molecules have an equal probability of forward or backward scattering. In contrast, in random porous structures, most pore walls are not parallel to the flow, and some pores are even oriented perpendicular to it. These geometric obstructions significantly enhance the probability of back-scattering and thus transport resistance (Fig. 1c).

To address this limitation, we establish an equivalent model: a cylindrical channel incorporating internal barriers (Fig. 1d), which more accurately captures the transport resistance inherent in random media. This model is characterized by four geometric parameters: total channel length (*L*), diameter (*d*), barrier spacing (*l*), and the constriction ratio (*ϕ*), with the latter defined as the ratio of the barrier opening to the channel cross-section. The total channel length is set equal to the thickness of the porous network. The constriction ratio is determined by analyzing the pore interconnectivity between successive layers of the network. Specifically, the network can be conceptualized as a stack of layers, each with a thickness equal to the pore length. Pores are assumed to be randomly distributed in each layer. Consequently, for a pore in one layer, the facing area in the adjacent layer comprises an open pore fraction of *ϕ* and a solid fraction of 1−*ϕ*. This implies that for a gas molecule approaching the interface, the probability of entering an open pore in the subsequent layer is equal to the porosity *ϕ*. To capture this transport mechanism, the constriction ratio of the barriers is set to the porosity of the porous network *ϕ* (Fig. 1d and Supplementary Fig. 1). This choice ensures that the barrier-channel model preserves the characteristic back-scattering behavior of random media while allowing analytical treatment of transport resistance.

Although the porous network is simplified, directly calculating the transmission probability through a straight channel with internal barriers remains mathematically challenging. To address this, we introduce the concept of transport resistance, *R*. A higher resistance corresponds to an increased probability of back-scattering and therefore a reduced net gas flux. The relationship between transmission probability, *α*, and transport resistance is defined as^25^

1 $$R=\frac{1}{\alpha }-1$$

In the limit where *α* = 1, all gas molecules entering the channel exit to the permeate side without back-scattering, yielding a resistance of *R* = 0. Conversely, as *α* approaches 0, which implies that most molecules scatter back to the feed side, the resistance diverges to infinity. For gas transport through a series of components, the total resistance is additive.

**Fig. 2:**
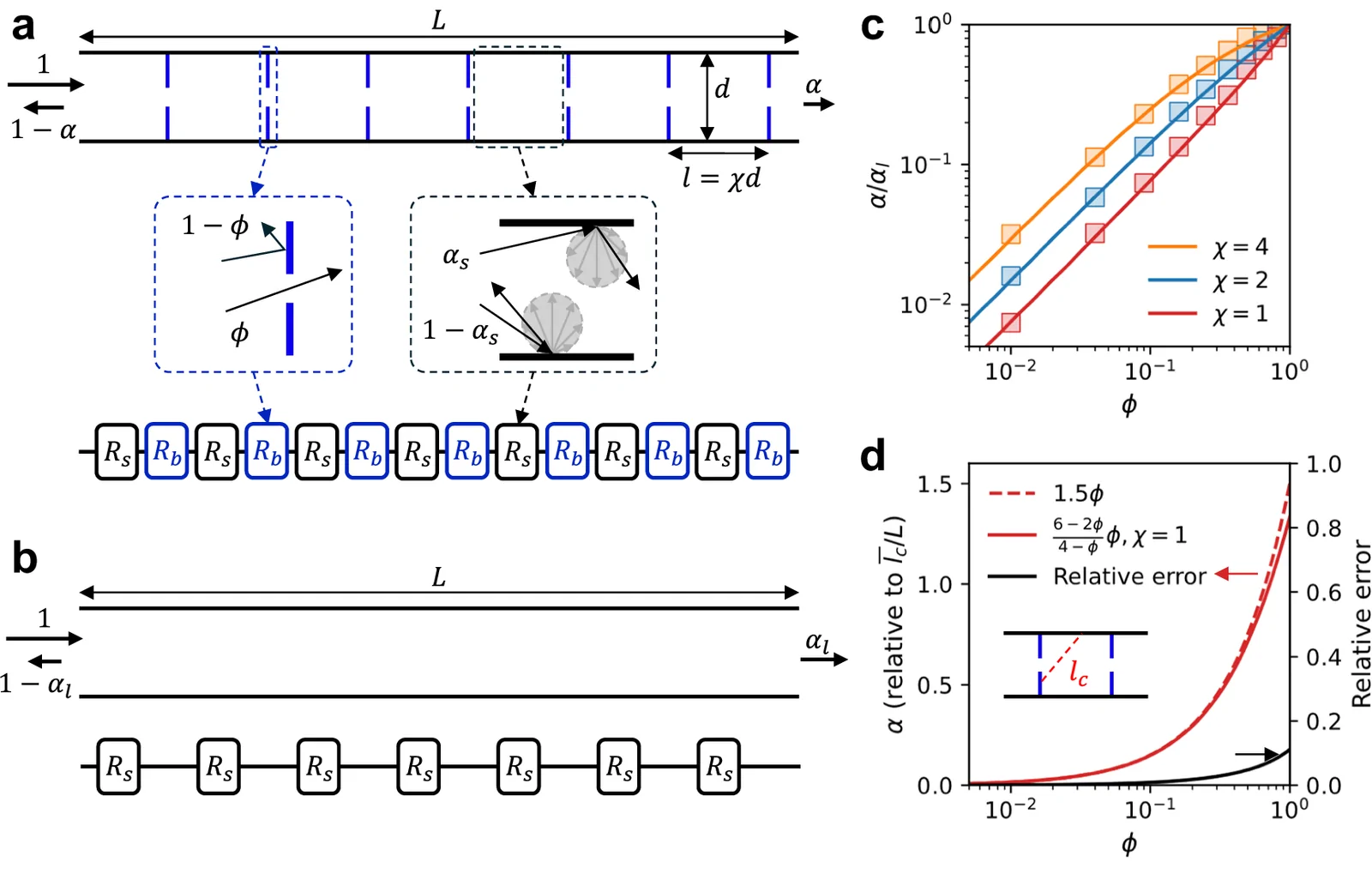
Transport resistance and transmission probability in channels with internal barriers. **a** Decomposition of transport resistance in a channel of length *L* and diameter *d* with periodic barriers spaced *l* apart. *R*_*b*_ denotes the resistance of a barrier, and *R*_*s*_ denotes the resistance of the short channel segment between adjacent barriers. The transmission probability *α* represents the likelihood that a gas molecule passes through the channel. The blue and black dashed boxes illustrate the back-scattering probabilities at the barrier, 1−ϕ, and within the channel segment, $1-{\alpha }_{s}$, respectively, both of which contribute to the total transport resistance. **b** Analogous decomposition of transport resistance for a barrier-free **c**hannel. **c** Transmission probability of channels with barriers, where the ratio of barrier spacing to channel diameter is *χ*. Data are normalized by the transmission probability of a barrier-free channel of the same length and diameter. Symbols represent MC simulation results, and solid lines represent predictions from Eq. (3). **d** Transmission probability of a channel with barrier spacing equal to the diameter (χ=1), plotted against the constriction ratio, ϕ. Values are normalized by the ratio of mean chord length to channel length. The red solid line represents Eq. (7), while the red dashed line shows the simplified approximation (1.5ϕ). The black solid line indicates the relative error of this simplification. The inset illustrates the chord with length *l*_*c*_, defined as the straight-line trajectory of a molecule between two successive collisions with the walls. Source data are provided as a Source Data file.

We decompose the channel with barriers into a series of discrete barriers and short channel segments located between them (Fig. 2a). For a barrier with a constriction ratio, ϕ, only a fraction ϕ of the gas molecules can pass through. Consequently, the transmission probability is ϕ, and the resistance is given by $\frac{1}{\phi }-1$. For the intermediate short channel segments, the transmission probability is defined as ${\alpha }_{s}$ and the corresponding resistance as ${R}_{s}$. Given a total channel length *L* and barrier spacing *l*, the system consists of *L*/*l* repeating units. Consequently, the total resistance of the channel is

2 $$R=\frac{L}{l}\left({R}_{b}+{R}_{s}\right)$$

A barrier-free channel of the same dimensions can be analogously modeled as a series of *L*/*l* short segments (Fig. 2b). Its resistance is given by $\frac{L}{l}{R}_{s}$, and its transmission probability is approximated by the classical equation $\frac{4d}{3L}$ ref. ^12^. By combining these resistance and transmission values with Eq. (1) and Eq. (2), we obtain the analytical expression for the transmission probability of channels with barriers:

3 $$\alpha=\frac{4d}{3L}\frac{3\phi \chi }{4-4\phi+3\phi \chi }$$

where the first term represents the transmission probability of a barrier-free channel of the same dimensions, and *χ* is defined as the ratio of barrier spacing to channel diameter (χ=l/d). To verify Eq. (3), MC simulations were performed to calculate the transmission probability through channels with varying *χ* and constriction ratios *ϕ* (see Methods for details). The analytical prediction from Eq. (3) shows excellent agreement with the MC simulation results (Fig. 2c).

In a random isotropic porous network, pore dimensions are statistically uniform in all directions. Based on this geometric uniformity, *χ* is assumed to be 1. Thus, Eq. (3) can be simplified to

4 $$\alpha=\frac{4d}{3L}\frac{3\phi }{4-\phi }$$

To eliminate the dependence on *d*, the mean chord length, $\bar{{l}_{c}}$, is introduced as a geometric substitute, defined as the average straight-line trajectory of a molecule between two successive wall collisions (inset of Fig. 2d), which can be determined from the pore structure or even a cross-section of the porous network. This value is governed by a rigorous expression^26^:

5 $$\bar{{l}_{c}}=\frac{4V}{S}$$

where *V* and *S* represent the volume and surface area of a representative channel unit, respectively. Applying this definition to the channel geometry establishes the relationship between $\bar{{l}_{c}}$ and *d*:

6 $$\bar{{l}_{c}}=\frac{\pi {d}^{3}}{\pi {d}^{2}+\frac{1}{2}\pi\left(1-\phi \right){d}^{2}}=\frac{2d}{3-\phi }$$

Consequently, Eq. (4) can be reformulated in terms of the mean chord length:

7 $$\alpha=\phi \frac{\bar{{l}_{c}}}{L}\frac{6-2\phi }{4-\phi }$$

which can be further approximated to a concise form:

8 $$\alpha=1.5\phi \frac{\bar{{l}_{c}}}{L}$$

The relative error introduced by this final simplification is minimal (Fig. 2d). For *ϕ* below 0.8, the error remains below 8.5%, and the accuracy improves further as *ϕ* decreases. This equation, Eq. (8), represents the key finding of this work, providing a concise law dependent only on three measurable structural properties: porous structure thickness, porosity, and mean chord length. Crucially, for random porous media, established stereological principles show that the 3D mean chord length and porosity are statistically equivalent to those measured from 2D cross-sections^27^. Therefore, transport properties can be predicted directly from 2D structural images, greatly simplifying characterization and enabling practical permeability estimation.

### Theory validation in random porous networks spanning a large porosity range

**Fig. 3:**
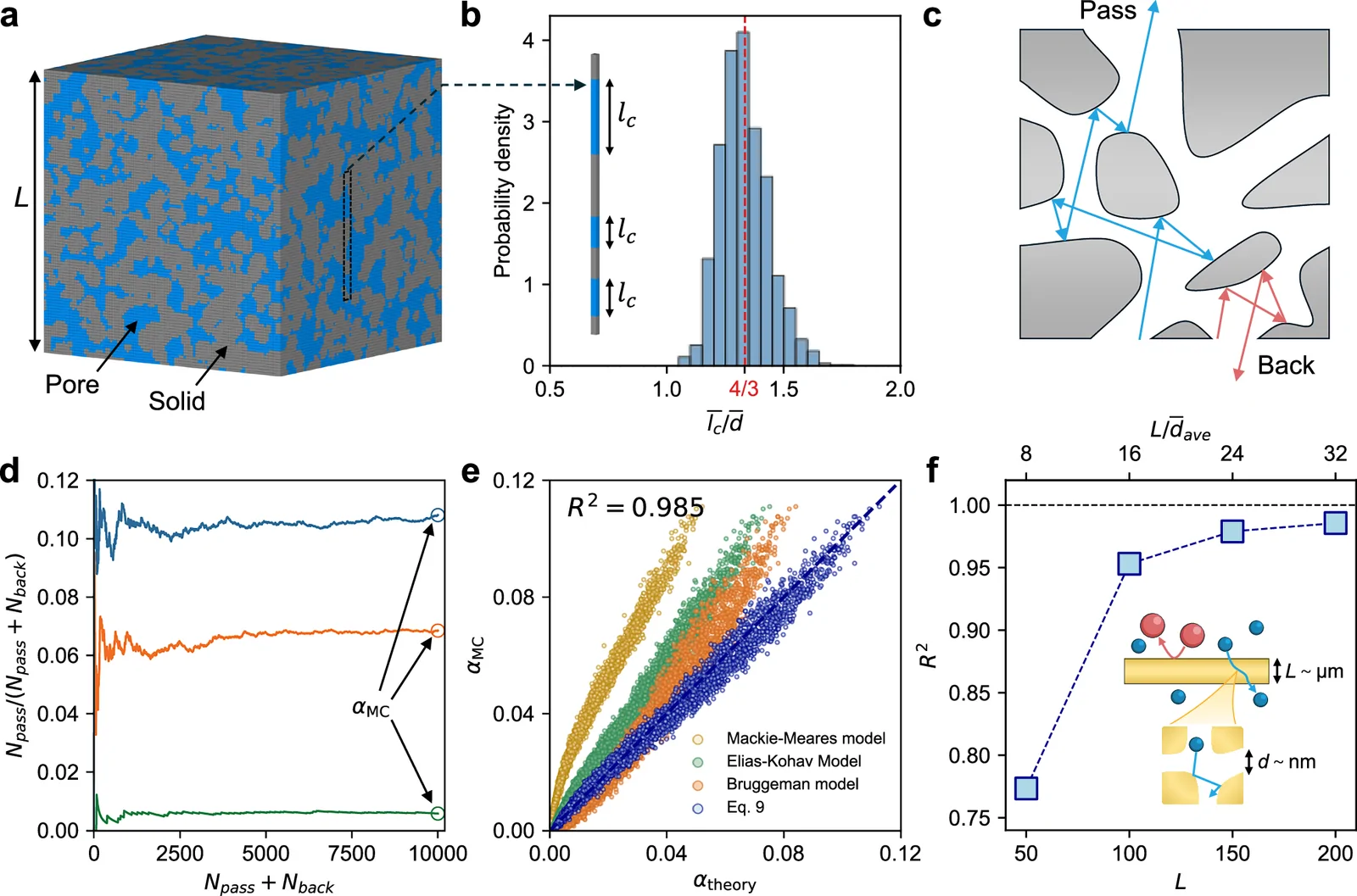
Verification of the theoretical framework by MC simulations. **a** Example of a randomly generated porous network with thickness *L*. Blue regions represent pore space, and gray regions denote the solid matrix. **b** Probability density function of the ratio between mean chord length and mean pore diameter for randomly generated networks. The inset illustrates the determination of chord length. **c** MC simulation of gas transport in the porous network, where gas molecules finally exit from the other side (blue trajectories) or return to the initial side (red trajectories). **d** Convergence of the transmission probability calculated by MC simulations for three porous networks with porosities of 0.16, 0.71, and 0.79. **e** Comparison between transmission probabilities obtained from MC simulations and theoretical predictions calculated using the proposed theory (Eq. (9)) and other tortuosity-based models. **f** The coefficient of determination (*R*^2^) between MC simulations and the proposed theory as a function of network size and the ratio of thickness to mean pore size, showing a convergence toward unity. The inset illustrates the high ratio of porous structure thickness to pore-size characteristic of gas separation membranes, confirming the applicability of our model to practical materials. Source data are provided as a Source Data file.

To validate the proposed theory, transmission probabilities through random porous networks were calculated using MC simulations (see Methods for details). First, random porous networks with varying porosities and thicknesses were constructed using a modified random sphere packing method (Fig. 3a and Supplementary Fig. 2). Since mean pore diameter ($\bar{d}$) is a more commonly used descriptor than mean chord length in materials science, the relationship between these two parameters was examined. For all networks, the ratio of mean chord length to mean pore diameter clusters narrowly around 4/3 (Fig. 3b). Substituting this relationship into Eq. (8) yields:

9 $$\alpha=2\phi \frac{\bar{d}}{L}$$

MC simulations were then carried out to evaluate the transmission probability from the feed to the permeate side through these networks (Fig. 3c). Gas molecules, treated as point particles, were randomly positioned at the porous network entrance with randomly assigned velocities directed into the network. In simulations, gas molecules followed Knudsen flow, moving in straight lines until colliding with solid surfaces and then diffusely reflecting according to Lambert’s cosine law^28^. Each gas trajectory either passed through the network or returned to the feed side. When a sufficiently large number of trajectories were simulated, the fraction of molecules passing through the porous network converged to a stable value representing the transmission probability. In this work, 10,000 trajectories were simulated for each network, which was sufficient to ensure convergence across all porosities (Fig. 3d). The transmission probability ${\alpha }_{{MC}}$ was calculated via MC simulations for 5000 porous networks with a thickness of 200 and porosities ranging from 0 to 0.8 (Supplementary Fig. 3).

The theoretical transmission probability, ${\alpha }_{{theory}}$, was calculated using Eq. (9) based on the mean pore size and porosity of these networks. When compared with other established tortuosity-based models (see Methods for details), our proposed framework demonstrates superior accuracy (Fig. 3e). The theoretical predictions show excellent agreement with the MC simulation results, achieving a coefficient of determination (*R*^2^) of 0.985 (Fig. 3e).

Porous networks with thicknesses ranging from 50 to 200 were compared (Fig. 3f and Supplementary Fig. 4). For each network size, the pore size was about 6.25, averaged over all simulated porous networks, yielding thickness-to-pore-size ratios ranging from 8 to 32. As this ratio increased, the structure became more homogeneous, and the *R*^2^ values approached unity (Fig. 3f and Supplementary Fig. 4). These results suggest that our theory is particularly well suited for homogeneous porous networks, typical of most isotropic porous materials, where the material thickness is significantly greater than the pore size (inset of Fig. 3f).

The current model assumes that gas molecules undergo fully diffuse reflection upon colliding with pore walls, which is the standard assumption of classical Knudsen theory. However, in atomically smooth straight channels like carbon nanotubes or graphene, specular reflection can lead to gas fluxes much higher than those predicted by classical Knudsen theory^8,16^. In previous work, specular reflection was considered to quantify this flux enhancement in straight channels^15^. For disordered porous media, however, internal structural barriers force molecules to backscatter regardless of the reflection mode. In addition, most materials typically exhibit atomic-scale roughness, which promotes diffuse gas reflection^29^. These factors substantially reduce flux enhancement associated with specular reflection. Therefore, the assumption of fully diffuse reflection is reasonable for such disordered porous systems (Supplementary Text S1 and Supplementary Fig. 5).

### Application to real porous materials: predicting membrane permeability and selectivity

**Fig. 4:**
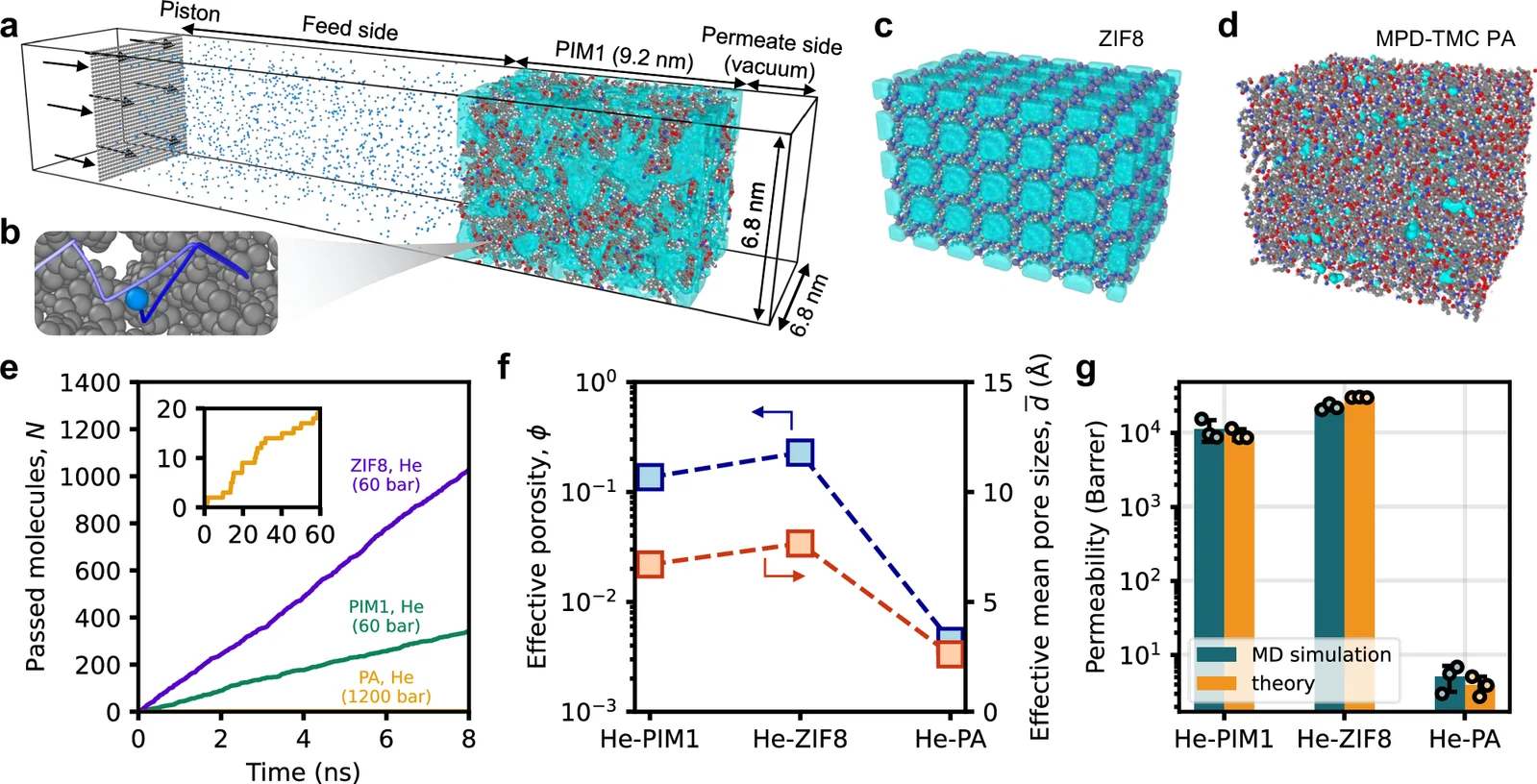
Gas permeation through real porous membrane materials. **a** The setup of NEMD simulations of helium gas permeation through PIM-1 porous materials from the feed side to the permeate side (vacuum), with feed-side pressure applied by a piston. Blue regions denote the pore space in PIM-1. **b** Helium trajectories inside PIM-1’s pore structure, illustrating Knudsen diffusion behavior. **c** The atomic and pore structure of ZIF-8. **d** The atomic structure and pore distribution in MPD-TMC polyamide (PA) membrane. **e** Number of helium atoms entering the permeate vacuum side as a function of time for the three membranes. **f** Effective porosity and effective mean pore size in PIM-1, ZIF-8, and PA experienced by helium atoms. **g** Helium permeability calculated by MD simulations and our theoretical model (Eq. (13)). Each data point represents an independent membrane sample. Error bars represent the standard deviation of three samples. Source data are provided as a Source Data file.

The proposed theory was validated using random porous networks generated by the random sphere packing method. However, real porous materials exhibit more complex and heterogeneous structures. To further assess the theory, representative porous membrane materials were constructed, and NEMD simulations (see Methods for details) were performed to evaluate gas permeability (Fig. 4a, b) and compare with theoretical predictions. In the NEMD setup, the porous membrane was placed between a gas feed side and a vacuum permeate side, creating a pressure difference that drove gas transport through the membrane.

Three representative membrane materials, PIM-1 (Fig. 4a), ZIF-8 (Fig. 4c), and MPD-TMC polyamide (PA) (Fig. 4d), were selected to span a broad range of porosities and structural order. Although random porous networks constitute the primary target of our theoretical framework, ZIF-8 was included as an additional exploratory test system. Its characteristic cavity–window architecture introduces well-defined internal geometric barriers, providing an opportunity to evaluate the physical assumptions underlying the barrier model in a highly ordered porous material.

Our theory describes Knudsen transport, in which gas molecules undergo diffuse reflections upon collision with pore walls. Helium was selected for the simulations because gases with stronger intermolecular interactions can exhibit significant adsorption and surface diffusion within the membrane, thereby obscuring direct comparisons with the Knudsen-based framework^30^. By contrast, helium interacts only weakly with membrane atoms and exhibits negligible surface adsorption (Fig. 4b). This assumption was further validated through simulations of helium transport in a well-defined slit pore formed by MPD-TMC PA membranes. In this system, the simulated gas flux agreed closely with the prediction of Knudsen theory and did not exhibit appreciable surface slip or localized density enhancements near the pore walls (Supplementary Fig. 6).

As helium permeated through these porous membrane structures, the number of gas molecules reaching the permeate side was counted over time (Fig. 4e), from which the gas permeability was calculated:

10 $${P}_{{MD}}=\frac{{dN}}{{dt}}\frac{L}{A\Delta p}$$

where *N* represents the number of permeating gas molecules over time *t*, *L* is the membrane thickness, *A* is the membrane cross-sectional area, and Δ*p* is the pressure difference between the feed and permeate sides. In this work, Δ*p* equals the feed-side pressure since the permeate side was maintained under vacuum. The feed-side pressure was set to 60 bar for PIM-1 and ZIF-8, and to 1200 bar for MPD-TMC PA to ensure adequate statistical sampling. The use of elevated pressures is a standard practice in molecular dynamics (MD) simulations^31,32^, and our previous work demonstrated minimal deviation between gas permeability obtained at 1200 bar and 60 bar^33^. Under these elevated pressure conditions, helium transport in these membranes remains in the Knudsen-dominated regime (Supplementary Text S2).

For each membrane, the effective porosity and mean pore size were calculated from the membrane configurations obtained under the corresponding NEMD pressure conditions using a probe with a diameter of 2.6 Å, corresponding to the kinetic diameter of helium (Fig. 4f). Based on the porosity, mean pore size, and membrane thickness, the transmission probability was predicted using our proposed theory (Eq. (9)). The transmission probability is defined as the ratio of the gas flux exiting the membrane at the permeate side to the gas flux entering the membrane from the feed side. According to kinetic theory, the gas flux entering the membrane from the feed side is given by ref. ^15^

11 $${\dot{n}}_{{in}}=\frac{\phi A\Delta p}{\sqrt{2\pi {RT}M}}$$

where ϕA represents the pore area on the membrane surface facing the feed side, *R* is the gas constant, *T* is the temperature, and *M* is the molar mass of the gas. At the interface between the feed side and the membrane, gas molecules flow both from the feed side into the membrane and from the membrane back to the feed side. We note that ${\dot{n}}_{{in}}$ represents the flux from the feed side into the membrane, not the net flux at this interface. Given the gas flux entering the membrane and the transmission probability, the gas flux entering the permeate side can be expressed as

12 $${\dot{n}}_{{out}}=\alpha {\dot{n}}_{{in}}=\frac{2\bar{d}{\phi }^{2}A\Delta p}{L\sqrt{2\pi {RT}M}}$$

The gas permeability is then given by:

13 $${P}_{{theory}}={\dot{n}}_{{out}}\frac{L}{A\Delta p}=\frac{2\bar{d}{\phi }^{2}}{\sqrt{2\pi {RT}M}}$$

Based on Eq. (10) and Eq. (13), gas permeabilities were obtained through both NEMD simulations and our proposed theory. The theoretical predictions were found to agree well with the MD simulation results (Fig. 4g). Since experimentally reported helium permeabilities span several orders of magnitude^34^, and the effective membrane thickness, a critical parameter for permeability calculations, remains uncertain^35^, direct comparisons with experimental permeability were not pursued.

When gases undergo Knudsen flow, their selectivity can be expressed according to classic Knudsen theory^36^:

14 $${S}_{A/B}=\sqrt{{M}_{B}/{M}_{A}}$$

where A and B denote two gas species. While Eq. (14) is widely used to verify Knudsen diffusion behavior, it is based on the assumption that the pore size is significantly larger than the kinetic diameters of the gas molecules. Under this assumption, the gas molecular size can be neglected, the molecules can be treated as point masses, and the effective pore size is the same for all gas species.

**Fig. 5:**
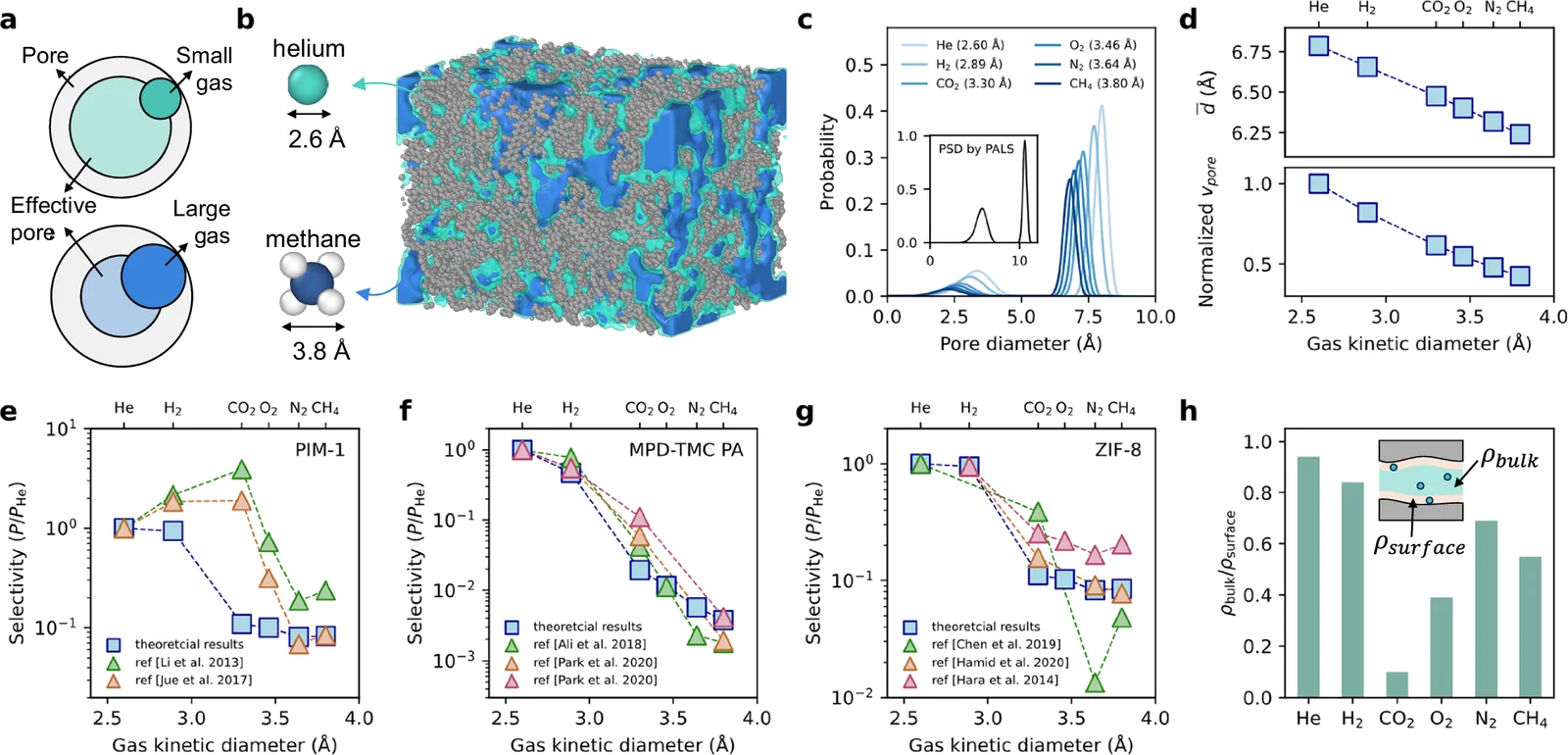
Gas selectivity predicted by the developed theory. **a** Schematic illustrating the effective pore size experienced by gases with small and large molecular sizes. **b** Accessible pore distribution in PIM-1 (6.8 × 6.8 × 9.2 nm^3^) for helium and methane molecules. **c** Effective pore-size distribution of PIM-1 for gases with different kinetic diameters calculated by Eq. (16). Inset: pore-size distribution of PIM-1 obtained from Positronium Annihilation Lifetime Spectroscopy (PALS)^39^. **d** Effective mean pore size and pore volume in PIM-1 for gases with different kinetic diameters. **e**–**g** Gas selectivity relative to helium ($P/{P}_{{{\rm{He}}}}$) in PIM-1^40,41^, PA^42,43^, and ZIF-8^34,44,45^ membranes calculated by our theory and by reported experimental results. **h** Ratio of gas density in the bulk pore region to that near the PIM-1 membrane atoms for gases with different kinetic diameters. The inset shows a schematic of the surface and bulk regions. Source data are provided as a Source Data file.

However, this assumption breaks down when pore sizes are comparable to gas molecular dimensions, a common situation in gas separation applications^37,38^. In such cases, the impact of gas size on the effective pore size must be considered by defining an effective pore size: ${d}_{{eff}}=d-{d}_{{gas}}$. Small molecules therefore access a larger pore space than larger ones (Fig. 5a), leading to different effective porosities. Incorporating this effect, and using Eq. (13) as the base permeability expression, we obtain the modified selectivity relation:

15 $${S}_{A/B}=\frac{{\bar{d}}_{A}{\phi }_{A}^{2}/\sqrt{{M}_{A}}}{{\bar{d}}_{B}{\phi }_{B}^{2}/\sqrt{{M}_{B}}}$$

Taking PIM-1 as an example, helium gas (2.6 Å) experiences a larger accessible pore space than methane (3.8 Å) (Fig. 5b). The mean effective pore size and porosity accessible to methane are smaller than those for helium, with the pore-size distribution skewed toward smaller values. The pore-size distribution is volume-weighted. When the effective pore size is ${d}_{{eff}}$, the accessible pore volume is scaled down by a factor of ${d}_{{eff}}^{3}/{d}^{3}$, yielding the effective pore-size distribution:

16 $${{PSD}}^{*}\left({d}_{{eff}}\right)={PSD}\left(d\right)\frac{{d}_{{eff}}^{3}}{{d}^{3}}$$

The pore-size distribution of PIM-1 from PALS measurements, as reported in the literature^39^, is shown in the inset of Fig. 5c. Based on Eq. (16), we obtained the effective pore-size distributions of PIM-1 for gases with different kinetic diameters: helium (2.6 Å), hydrogen (2.89 Å), carbon dioxide (3.3 Å), oxygen (3.46 Å), nitrogen (3.64 Å), and methane (3.8 Å). From these distributions, effective mean pore size and normalized pore volume were determined (Fig. 5d). These values were then used to calculate gas selectivity relative to helium via Eq. (15), and compared with experimental results^40,41^.

For PIM-1, the theoretical selectivities for nitrogen/helium and methane/helium align well with experimental data, though carbon dioxide/helium selectivity falls below measured values (Fig. 5e). MPD-TMC PA^42,43^ and ZIF-8^34,44,45^ showed similar agreement (Fig. 5f, 5g, and Supplementary Fig. 7). To explain why the reported carbon dioxide permeability exceeds theoretical predictions, gas transport mechanisms were analyzed. Gas density in PIM-1 was analyzed at different distances from membrane atoms (inset of Fig. 5h). Surface regions (2–4 Å) and bulk regions (>4 Å) were defined accordingly (Supplementary Fig. 8). For ballistic Knudsen transport, surface and bulk densities should be similar, whereas strong surface adsorption results in much higher surface density. The bulk-to-surface density ratio was calculated for each gas. Helium and hydrogen show ratios of 0.94 and 0.84, respectively, close to 1 and consistent with Knudsen flow. Nitrogen, oxygen, and methane exhibit ratios of 0.69, 0.39, and 0.55, suggesting a combination of Knudsen transport and surface adsorption. Carbon dioxide shows a ratio of approximately 0.10, indicating strong surface adsorption and possible condensation rather than ballistic transport, as also reported in other studies^40^. These results explain why our theory can capture gas selectivity in nanoporous materials for weakly adsorbing gases, but fails for gases with strong adsorption.

## Discussion

In this work, we extended the classical Knudsen theory to random porous networks, providing a practical and accessible tool for predicting gas permeability and selectivity in nanoporous materials. By simplifying complex porous networks into equivalent circular channels with internal barriers, we rigorously derived simple analytical expressions that capture the essential physics of gas transport in disordered media. The resulting permeability equation requires only two measurable structural parameters: mean pore size (or mean chord length) and porosity. MC simulations across randomly generated networks with porosities from 0 to 0.8 validated the theoretical framework, demonstrating better agreement compared to existing models. When the network thickness exceeds approximately 32 times the mean pore size, a regime representative of most gas separation membranes, the prediction accuracy achieves *R*^2^ = 0.985. NEMD simulations of gas permeation through PIM-1, ZIF-8, and MPD-TMC PA membranes further confirmed the applicability of the theory to real porous materials. By accounting for effective pore size, we further extended the framework to predict gas selectivity in materials with sub-nanometer pores, yielding good agreement with experimental data for weakly adsorbing gases.

Our work presents two key theoretical contributions. First, it extends the classical Knudsen theory from well-defined channels to random porous networks, significantly broadening the applicability of Knudsen theory, as most nanoconfined gas flows in practice occur in disordered complex porous networks. Second, the Knudsen theory was extended to porous networks with pore sizes comparable to gas molecules, enabling quantitative prediction of the ultrahigh gas selectivity in sub-nanometer porous materials, which previously was attributed to molecular sieving without a rigorous quantitative description.

Our theoretical framework enables prediction of gas permeability and selectivity using only two readily accessible parameters: mean pore size (or mean chord length) and porosity, which can be obtained from experiments or calculated from atomic structures. For random porous structures, the mean chord length and porosity in a 2D cross-section are statistically equivalent to their 3D counterparts, eliminating the need for full 3D pore reconstruction. Consequently, the parameters determining gas permeability can be directly obtained from 2D cross-sections, which are significantly easier to acquire than full 3D structures. Furthermore, building on the accurate prediction of gas flux, the framework enables inverse design. Given target permeability and selectivity, the required pore size and porosity can be analytically determined, providing an accessible tool for materials screening and design in gas-involved applications.

Our simplification of disordered porous networks into a barrier-channel model represents a conceptual departure from existing approaches. Many theoretical frameworks for gas or liquid transport in porous networks focus on the molecular transport path length, simplifying the porous structure into an equivalent straight channel having the same effective path length. In contrast, our framework treats the porous medium as a stack of layers along the flow direction. Transport resistance is evaluated between adjacent layers, and the porous structure is represented as a straight channel with the same thickness as the medium but containing internal barriers. These two approaches require accurate descriptions of the transport path length (characterized by tortuosity) and the barrier properties (characterized by the barrier constriction ratio), respectively, to close the models. For some theories, tortuosity must be estimated empirically, as in the Kozeny–Carman model^46^. In contrast, the barrier constriction ratio represents the probability that a molecule finds an open passage to the next layer and is directly determined by porosity, requiring no empirical fitting for random, isotropic porous media.

The barrier framework is also more rigorous for describing Knudsen transport, particularly when reflection mechanisms are considered. When gas molecules enter a porous network from the feed side, backflow arises from two distinct sources: (i) diffuse reflection at pore walls and (ii) geometric barriers that are not parallel to the flow direction. Tortuosity-based simplifications account only for the increased transport path length and neglect the contribution of these geometric barriers. Consequently, in the theoretical limit of purely specular reflection at pore walls, the barrier model correctly predicts a transmission probability smaller than unity due to geometric obstruction. By contrast, tortuosity-based models fail to capture this intrinsic source of resistance and predict a transmission probability of unity.

In porous networks, structural features such as pore connectivity and the presence of dead-end pores significantly influence gas transport. Explicitly parameterizing these pathway-dependent properties substantially complicates porous gas flow models. Our equivalent strategy, which divides the network into consecutive stacked layers and statistically evaluates transmission between adjacent layers, avoids the need to explicitly define these complex variables. Nevertheless, the physical effects of connectivity and dead ends are implicitly captured by the system’s porosity and mean pore size. In a random isotropic network, these two variables collectively determine the pore number density. At a given mean pore size, a higher porosity corresponds to a greater number of pores and a higher likelihood of pore interconnection and accessible transport pathways, whereas lower porosity increases the probability of isolated or dead-end pore regions. Thus, the effect of connectivity and dead-end pores is implicitly incorporated through the ensemble-average transport resistance represented by porosity and mean pore size.

Despite its broad applicability, the current framework has several limitations. First, surface diffusion and capillary condensation can contribute substantially to gas transport in nanoporous materials, particularly for strongly adsorbing gases. As the present theory focuses solely on Knudsen diffusion, it does not capture these mechanisms. Consequently, the framework is applicable to weakly adsorbing gases such as helium and hydrogen, as well as to gas transport carried out at elevated temperatures where adsorption effects are suppressed. Second, our framework and the simplification of *χ* = 1 assume fully random, isotropic porous networks. For highly ordered materials such as aligned one-dimensional pores, classical Knudsen theory is sufficient. For anisotropic networks with randomly oriented, non-spherical pores, *χ* should be treated as an independent structural parameter. Third, when pore sizes approach molecular dimensions, thermal motions of membrane atoms create dynamic pores that can influence gas transport. Although our results demonstrate good predictive capability for such membranes, the present framework does not explicitly account for these dynamic pore fluctuations. Fourth, for porous media with atomically smooth walls, although such systems are uncommon in practical applications, the effects of specular reflection on gas flux should be considered explicitly. Fifth, our model characterizes the pore-size distribution using only the mean pore size. Although the mean pore size is obtained by integrating over the full pore-size distribution, other distributional features such as the distribution width are not explicitly accounted for.

## Methods

### MC simulation of gas transport in a porous network

MC simulations were conducted to calculate the transmission probability of gas through porous networks. To construct the networks, we adopted an approach similar to the random sphere packing model, but allowed the spheres to overlap to generate more general porous structures. A periodic simulation box was first defined, within which a random number of spheres with randomly assigned radii were placed at random positions, permitting overlap (Supplementary Fig. 2). The regions not occupied by any spheres were treated as pores, while the remaining regions represented the solid matrix. We selected one axis of the simulation box as the flow direction, defining the two opposite faces along this axis as the feed and permeate sides. All lengths in the MC simulations are given in dimensionless units, since the transmission probability depends only on the ratio of network thickness to pore size.

For each porous network, 10,000 MC simulations were performed. In each simulation, a particle treated as a point without size was randomly placed within the pore region at the feed-side interface and assigned an initial velocity direction towards the porous network following Lambert’s cosine law. The particle then moved with a step length of 0.1 along this direction. Its position was updated iteratively until the next step intersected a solid region (inside one of the spheres). When this occurred, the step length was reduced by half until it became smaller than 0.001, at which point a collision with the solid surface was considered to occur. The surface normal at the collision point was then computed, and a new velocity direction was generated according to Lambert’s cosine law to simulate diffuse reflection. The particle then continued its ballistic motion until the next collision. During the simulation, each step was checked to determine whether the particle had returned to the feed side or reached the permeate side. If either condition was met, the trajectory was terminated. After 10,000 trajectories, the transmission probability was obtained as the fraction of particles that reached the permeate side. The flowchart of the MC simulations is shown in Supplementary Fig. 9.

To validate the procedure, we generated several well-defined cylindrical channels with different aspect ratios and calculated their transmission probabilities using these MC simulations. The transmission probability of these channels can be expressed by the following equation^14^,

17 $${\alpha }_{K}^{{circ}}=1+\frac{{\chi }^{2}}{4}-\frac{\chi \sqrt{{\chi }^{2}+4}}{4}-\frac{{\left[\left(8-{\chi }^{2}\right)\sqrt{{\chi }^{2}+4}+{\chi }^{3}-16\right]}^{2}}{72\chi \sqrt{{\chi }^{2}+4}-288{\mathrm{ln}}\left(\chi+\sqrt{{\chi }^{2}+4}\right)+288{\mathrm{ln}}2},$$

where *χ* denotes the ratio of channel length *L* to its radius *d*/2. As *χ* tends toward infinity, Eq. (17) converges to the classical expression proposed by Knudsen^12^,

18 $${\alpha }_{K}^{{circ}}=\frac{4d}{3L}.$$

The MC results show good agreement with Eq. (17) across various aspect ratios and converge to Eq. (18) when the channel length is much larger than the radius (Supplementary Fig. 10). Since the thickness of most nanoporous membranes is several orders of magnitude greater than the mean pore size, Eq. (18) was adopted in this work.

### Tortuosity-based models for porous Knudsen flow

In the study of porous Knudsen flow, such as vapor transport through hydrophobic polymer membranes in membrane distillation, a common approach to estimating gas transmission probability is to introduce the tortuosity factor *τ*. Tortuosity is defined as the ratio of the actual diffusion path length to the thickness of the porous network. By simplifying the porous network into an equivalent capillary model with a mean pore diameter of d and an effective path length of τL, the transmission probability can be estimated as

19 $$\alpha=\frac{4d}{3\tau L}$$

Several empirical models are widely used to estimate tortuosity based on porosity, such as the Mackie-Meares^47^, Elias-Kohav^48^, and Bruggeman^49^ models:

20 $${\tau }_{{{\rm{Mackie}}}-{{\rm{Meares}}}}=\frac{{\left(2-\phi \right)}^{2}}{\phi }$$

21 $${\tau }_{{{\rm{Elias}}}-{{\rm{Kohav}}}}=\frac{1}{\phi }$$

22 $${\tau }_{{{\rm{Bruggeman}}}}=\frac{1}{\sqrt{\phi }}$$

### MD simulation of gas transport in nanoporous materials

MD simulations were performed to examine the applicability of our theory to real porous materials. Three representative gas separation membranes were selected as examples: PIM-1, MPD-TMC polyamide, and ZIF-8.

The PIM-1 membrane was composed of 16 polymer chains, each containing 32 repeating units. The PA membrane was created following our previous work^33^, consisting of 972 TMC and 1458 MPD monomers, with a cross-linking degree of 0.83. Both the PIM-1 and PA membranes were placed in boxes periodically along the X and Y directions, with dimensions of 6.8 nm × 6.8 nm × 20 nm. Each membrane was relaxed for a 21-step equilibration scheme under pressure applied by two pistons placed on both sides along the membrane’s Z direction^50^. After relaxation, the pistons were removed, yielding final membrane dimensions of 6.8 × 6.8 × 9.2 nm^3^ for the PIM-1 membrane and 6.8 × 6.8 × 8.8 nm^3^ for the PA membrane. The ZIF-8 membrane was constructed by replicating the unit cell 4 × 4 × 6 times along the X, Y, and Z directions, resulting in a final membrane size of approximately 6.8 × 6.8 × 10.2 nm^3^.

After constructing the membranes, a feed region containing helium gas and a permeate region under vacuum were placed on the two sides of each membrane. The gas pressure on the feed side was applied using a piston composed of a carbon atom layer with a lattice spacing of 0.2 nm. Driven by this pressure, gas molecules permeated from the feed side through the membrane to the permeate side. Once the gas entered the permeate region, it was removed to maintain vacuum conditions. The number of gas molecules inside the membrane was monitored as an indicator of permeation progress. This number increased as gas entered the membrane and reached a plateau once steady-state permeation was established. The number of gas molecules reaching the permeate side was then recorded, and the permeability was calculated by dividing this number by the counting time, membrane cross-sectional area, and pressure difference between the two sides, and multiplying by the membrane thickness. For the PIM-1 and ZIF-8 membranes, the feed-side pressure was maintained at 60 bar, and the counting time was 8 ns. For the PA membrane, due to its much lower gas flux, the feed-side pressure was increased to 1200 bar, and the counting time was extended to 60 ns to obtain sufficient data for permeability evaluation. The permeation simulations were conducted at 300 K.

The OPLS-AA force field^51^ was used for PIM-1, PA, and helium, with the parameters for PIM-1 and PA generated using LigParGen^52^. The force field parameters for ZIF-8 were adopted from the literature^53^. Periodic boundary conditions were applied in the X and Y directions. Atomic trajectories were integrated using the Velocity-Verlet algorithm with a 1 fs time step. All simulations were carried out in the canonical (NVT) ensemble using LAMMPS^54^.

The pore-size distribution and porosity were calculated by Zeo++^55^, with the probe size set to the kinetic diameter of the target gas. The mean pore size was determined by integrating the pore-size distribution weighted by the pore diameter across the entire pore-size range: $\bar{d}={\int }_{0}^{\infty }d\bullet P{SD}(d){{\rm{d}}}d$.

## Supplementary information

Supplementary Information

Transparent Peer Review file

## Source data

Source Data

## Data Availability

Source data are provided with this paper.
