# Supplementary material for: A generalized Knudsen theory for gas transport in disordered porous materials
Source: Nat Commun. 2026 Jul 16;17:8719. doi: 10.1038/s41467-026-75656-8 (PMC13490521; doi:10.1038/s41467-026-75656-8)
Supplement: Supplementary file 1 [file 41467_2026_75656_MOESM1_ESM.pdf]

Supplementary Information for

A generalized Knudsen theory for gas transport in disordered porous materials

Jianhao Qian [a], Ruoyu Wang [a] and Menachem Elimelech [a,b,c,*]

[a] Department of Civil and Environmental Engineering, Rice University, Houston, TX, 77005, USA

[b] Department of Chemical and Biomolecular Engineering, Rice University, Houston, TX, 77005, USA

[c] Rice Center for Membrane Excellence (RiCeME), Rice University, Houston, TX, 77005, USA

[*] Email: menachem.elimelech@rice.edu

Text S1. Effect of Maxwell reflection coefficient on gas transmission in disordered porous networks

The mode of gas reflection at the pore wall has a strong influence on gas transport, and is commonly described by the Maxwell reflection coefficient, f, where a gas molecule colliding with the wall undergoes diffuse reflection with probability f and specular reflection with probability $1 - f$ (Fig. S5). The derivation in this work assumes fully diffuse reflection ($f = 1$). Below we examine the deviation arising from applying the $f = 1$ assumption when $f < 1$.

In ultra smooth channels such as carbon nanotubes, graphene channels, and hBN channels, specular reflection dominates (f approaching 0), producing gas fluxes much larger than those predicted by classical Knudsen diffusion[1,2]. However, disordered porous media differ from ultra smooth straight channels in two ways. First, in straight channels, specular reflection allows molecules to maintain their forward momentum and travel toward the permeate side. In disordered porous networks, structural barriers and pore constrictions can redirect molecules back toward the feed side regardless of whether the gas-wall reflection is diffuse or specular. Therefore, the main source of flux dissipation in disordered porous networks with low porosity is the internal structural resistance, rather than diffuse gas-wall reflection alone. Second, most disordered porous materials have atomically rough internal surfaces. Such roughness brings the Maxwell reflection coefficient close to unity, making the fully diffuse reflection assumption reasonable. For example, helium on glass, an amorphous surface that is more representative of PIM-1 and MPD-TMC polyamide than atomically smooth wall, has a reported momentum accommodation factor of 0.935, which corresponds closely to f in the Maxwell reflection model[3].

To quantify the influence of f on transport in disordered porous networks, we performed additional Monte Carlo simulations using the porous networks generated by the same method as in Fig. 3a. The Maxwell reflection coefficient was introduced by assigning diffuse reflection with probability f and specular reflection with probability $1 - f$ upon each gas-solid collision. For each of 800 generated porous structures, we calculated the transmission probability α_{MC} at $f = 0, 0.2, 0.4, 0.6, 0.8$, and 1.0. The enhancement caused by specular reflection was quantified as $\alpha_{MC}(f) / \alpha_{MC}(f = 1)$.

The results show that significant enhancement appears only when f approaches 0, corresponding to nearly specular reflection (Fig. S5). When f is close to unity, as expected for most atomically rough surfaces, the deviation from the purely diffuse Knudsen-type assumption is acceptable given the simplicity of the model.

Text S2. Collision-based assessment of the Knudsen-dominated regime

To examine whether the elevated pressures used in the NEMD simulations could induce non-Knudsen transport, we evaluated the relative importance of gas-wall and gas-gas collisions directly from the NEMD trajectories. For disordered porous materials, estimating a conventional Knudsen number using a single characteristic pore size is not straightforward because of the heterogeneous pore geometry. Therefore, we used a collision-based analysis to directly assess whether gas-wall collisions remain dominant over gas-gas collisions.

Specifically, membrane configurations were extracted from the NEMD simulations. For each helium atom located inside the membrane, we calculated its pairwise van der Waals interaction energy with other helium atoms and with membrane atoms. A collision was identified when the pairwise van der Waals interaction entered the repulsive regime, corresponding to a positive van der Waals pair energy. Thus, a He-He collision was counted when a helium atom had a positive van der Waals pair energy with another helium atom, while a He-solid collision was counted when it had a positive van der Waals pair energy with any membrane atom. The analysis was performed only for helium atoms located in the membrane interior, excluding those within 1 nm of the membrane boundaries.

We then calculated, for each helium atom, the probability of experiencing He-solid repulsive contacts and the probability of experiencing He-He repulsive contacts. The ratio of these two probabilities was used to quantify the relative importance of gas-wall and gas-gas collisions. For PIM-1 at a feed-side pressure of 60 bar, the gas-wall collision probability was 23.5 times higher than the gas-gas collision probability. For ZIF-8 at 60 bar, this ratio was 13.8. For MPD-TMC PA at 1200 bar, the ratio was still 6.0. These results show that gas-wall collisions remain dominant in all three membranes, even under elevated pressures. Therefore, the simulated transport remains in the Knudsen-dominated regime rather than transitioning to viscous-flow regime.

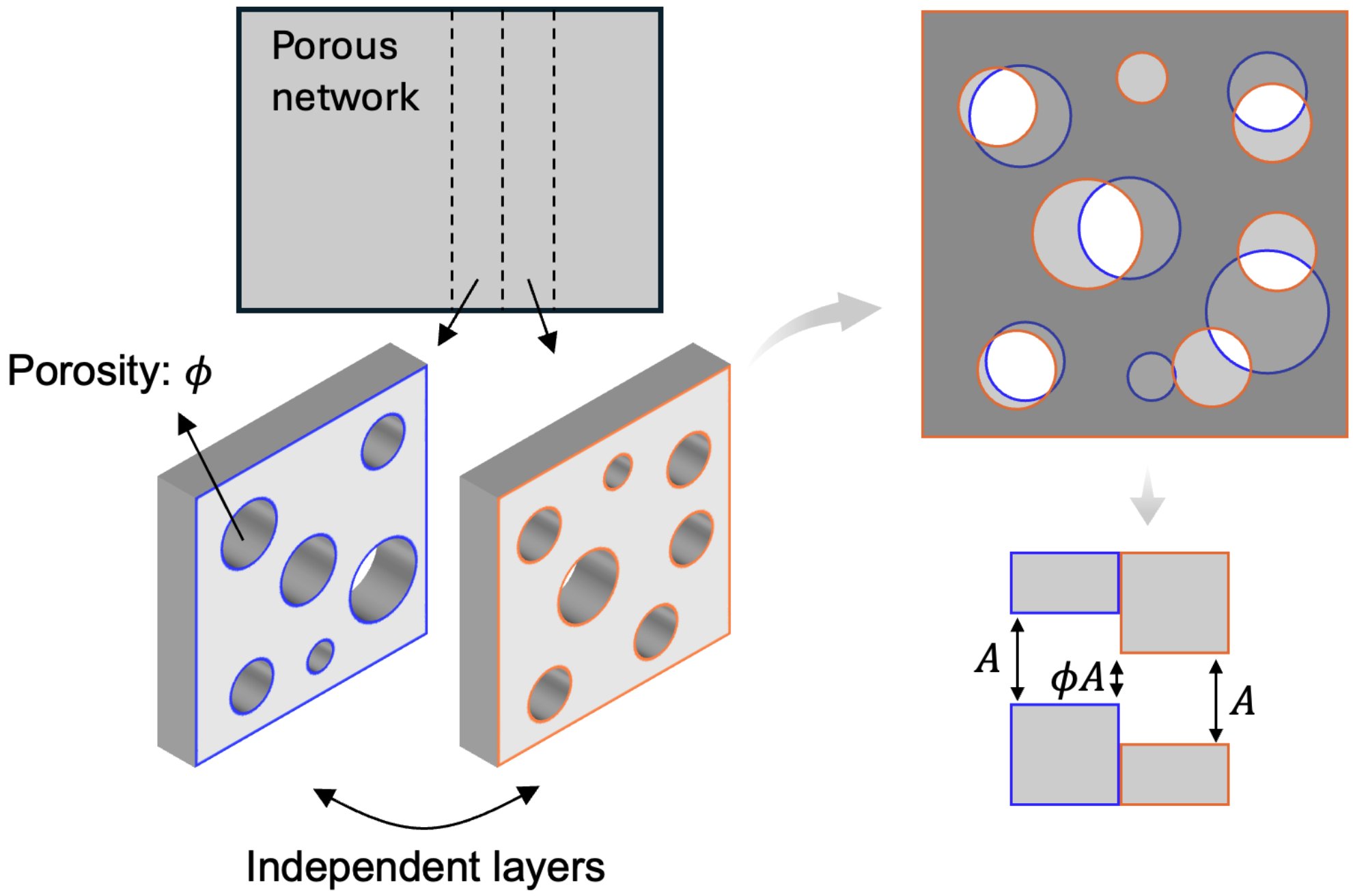

Fig. S1. Equivalence of the barrier constriction ratio of the 1D channel model as the porous network porosity.

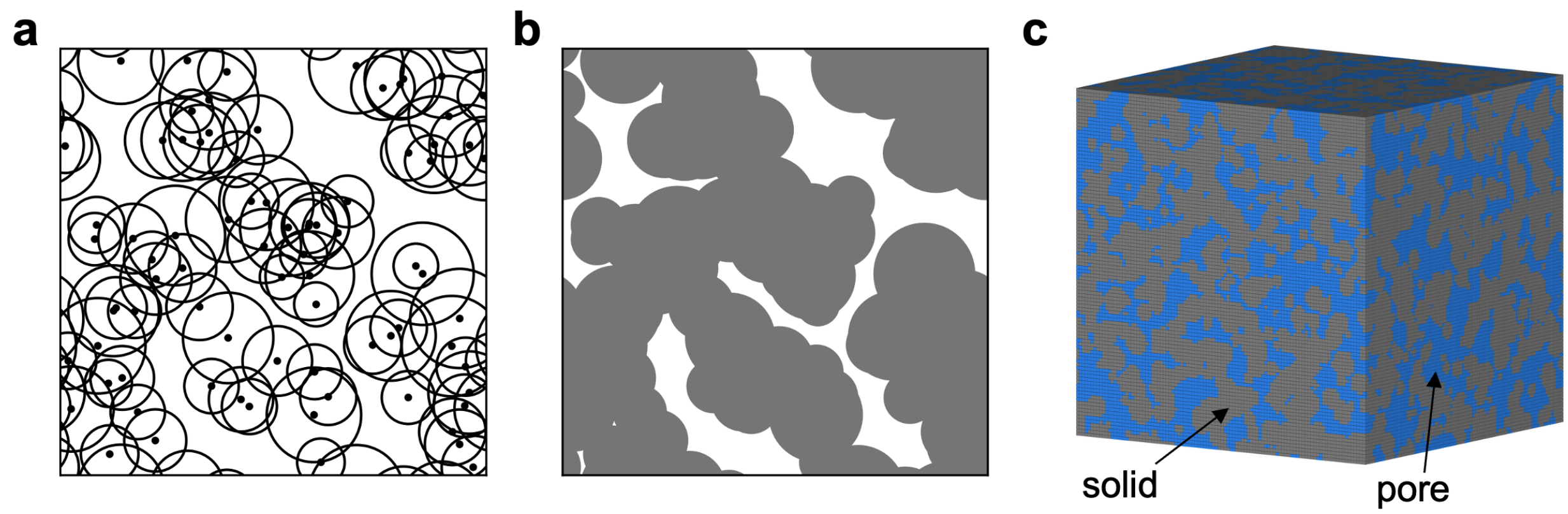

Fig. S2: Generation of a porous network. a Randomly distributed spheres with varied radius in a periodic box of defined size. **b** Definition of the solid area inside the spheres and the pore area outside the spheres defined in (**a**). **c** 3D view of the generated network, where the gray area represents the solid phase and the blue area represents the pores.

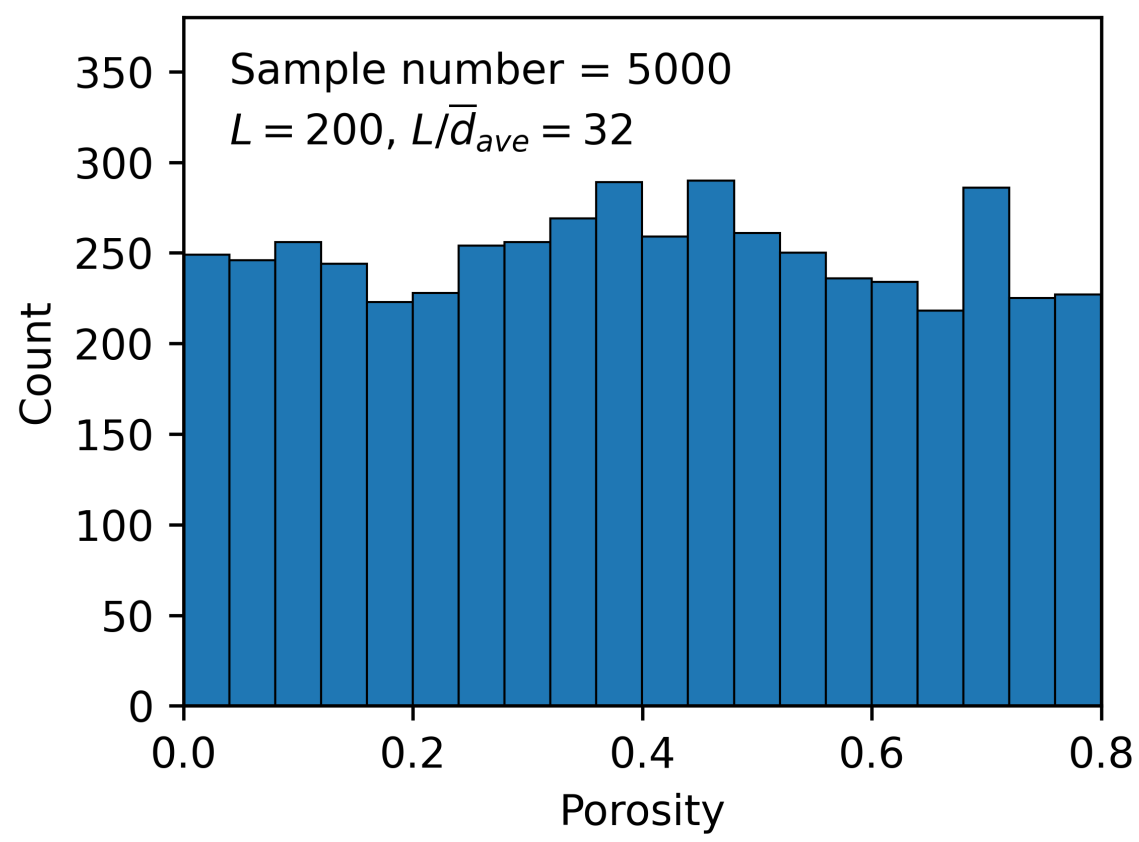

Fig. S3. Distribution of porosity in 5000 randomly generated porous network samples with a box thickness of L = 200 and a ratio of thickness to pore size equal to 32. The nearly uniform distribution confirms the statistical representativeness of the generated porous structures. Source data are provided as a Source Data file.

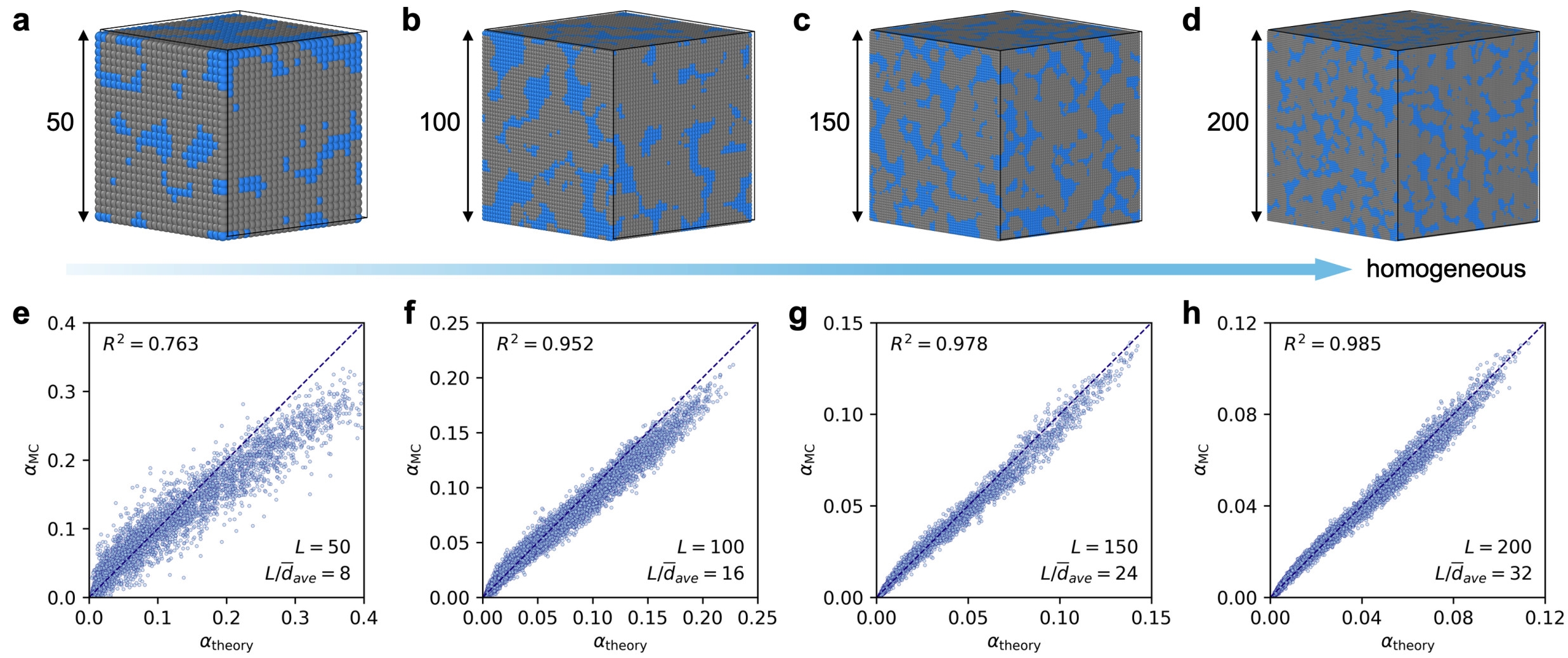

Fig. S4. Comparison between MC simulation results and theoretical predictions for porous networks with different sizes. a-d Randomly generated porous networks with box lengths L = 50, 100, 150, and 200, respectively. Blue regions represent pore spaces, and gray regions denote solids. **e-h** Correlation between the transmission coefficients from MC simulations and theoretical model for different box size. The increase in box size leads to higher ratios of box size to mean pore diameter, resulting in more homogeneous pore structures and stronger consistency between the transmission probabilities obtained from Monte Carlo simulations and theoretical predictions, with the R-squared value increasing from 0.763 to 0.985. Source data are provided as a Source Data file.

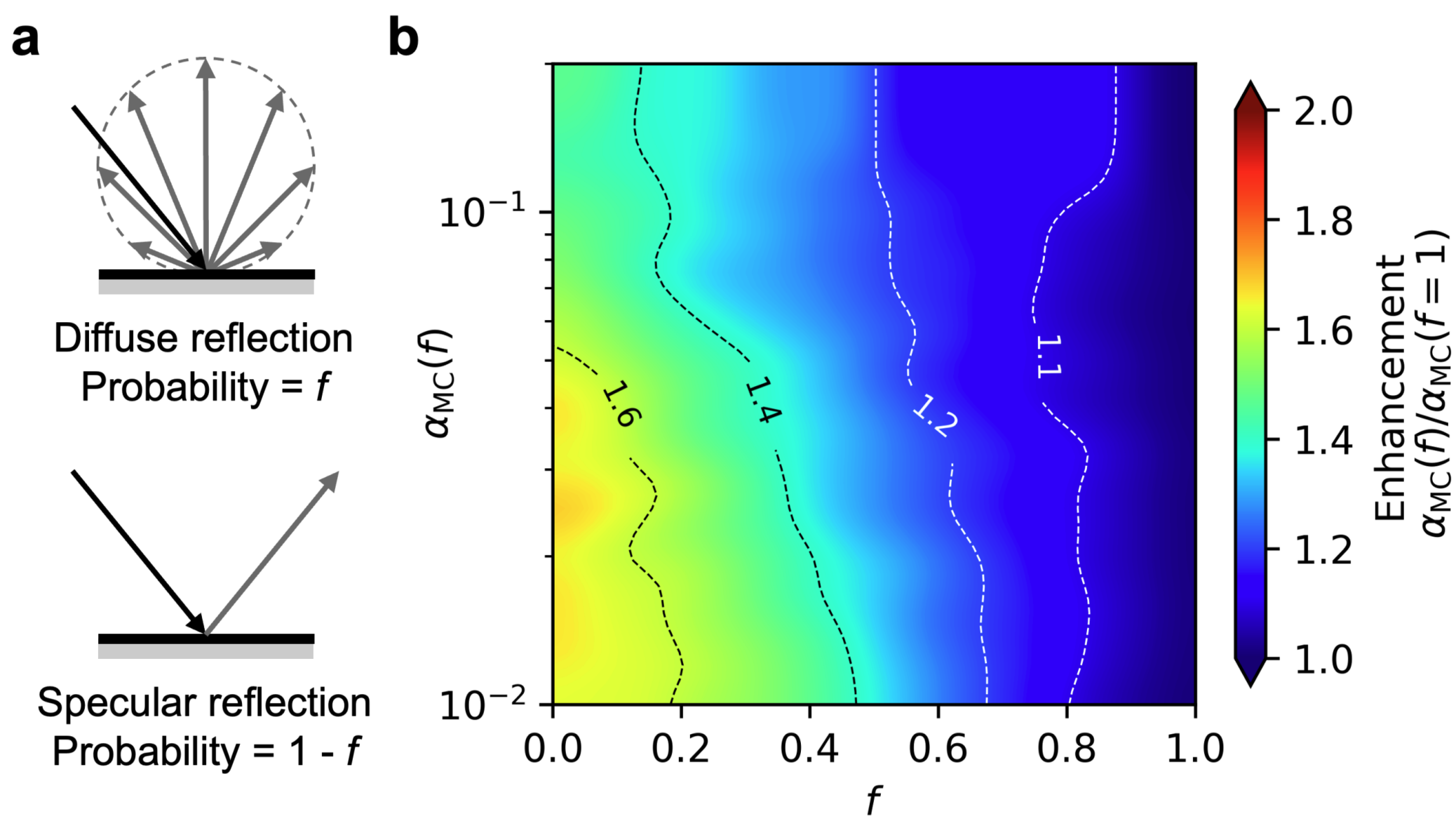

Fig. S5. Effect of the Maxwell reflection coefficient on transmission probability enhancement in porous networks. a Definition of the Maxwell reflection coefficient, f. When a gas molecule collides with a solid surface, it undergoes diffuse reflection with probability f and specular reflection with probability $1 - f$. **b** Transmission probability enhancement, $\alpha_{MC}(f)$ / $\alpha_{MC}(f = 1)$, calculated from Monte Carlo simulations. Each data point represents the transmission probability $\alpha_{MC}(f)$ of one porous network at a given diffuse-reflection probability f. The enhancement is obtained by normalizing this value by $\alpha_{MC}(f = 1)$, calculated for the same porous network under fully diffuse reflection. The contour map was constructed from 4800 data points, based on 800 porous network structures with $L = 200$, each evaluated at $f = 0$, 0.2, 0.4, 0.6, 0.8, and 1.0. Dashed lines denote representative enhancement levels. Source data are provided as a Source Data file.

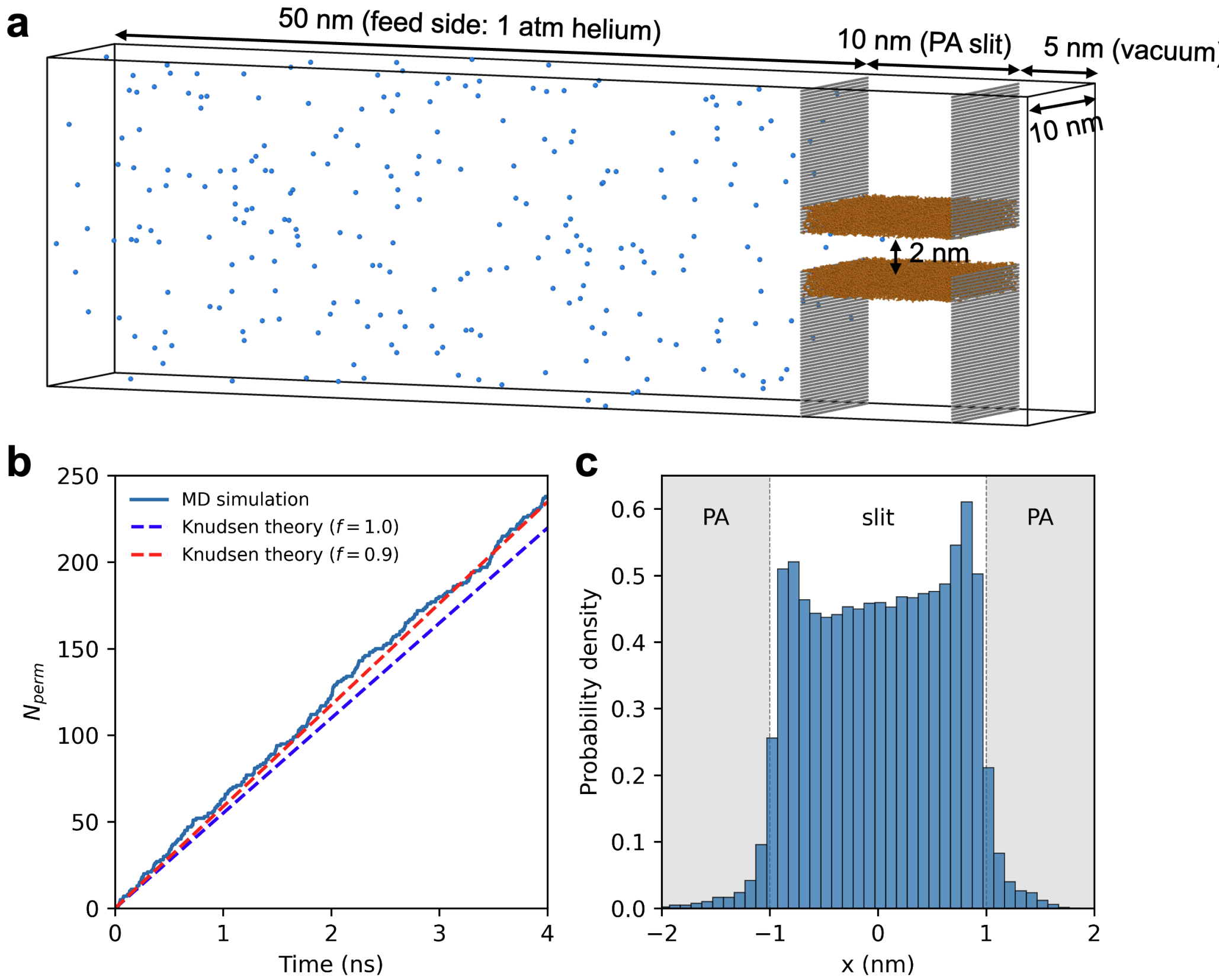

Fig. S6. Helium permeation through a polyamide slit and validation of the Knudsen model. **a** Setup for nonequilibrium molecular dynamics simulations of helium gas permeation through a slit 2 nm wide and 10 nm long, formed by two MPD-TMC PA sheets. Monatomic carbon sheets separate the feed side from the vacuum permeate side, ensuring that helium gas only passes through the PA slit. Helium atoms are colored blue and visually enlarged, PA membranes are dark orange, and carbon sheets are black. **b** The number of helium atoms permeating through the PA slit over time, compared with the Knudsen theory assuming fully diffuse reflection ($f = 1$) and partial diffuse reflection[4] ($f = 0.9$), where gas molecules colliding with the wall have a f probability of diffuse reflection and a $1 - f$ probability of specular reflection. **c** The density profile of helium gas in the slit along the normal direction of the PA membrane. Source data are provided as a Source Data file.

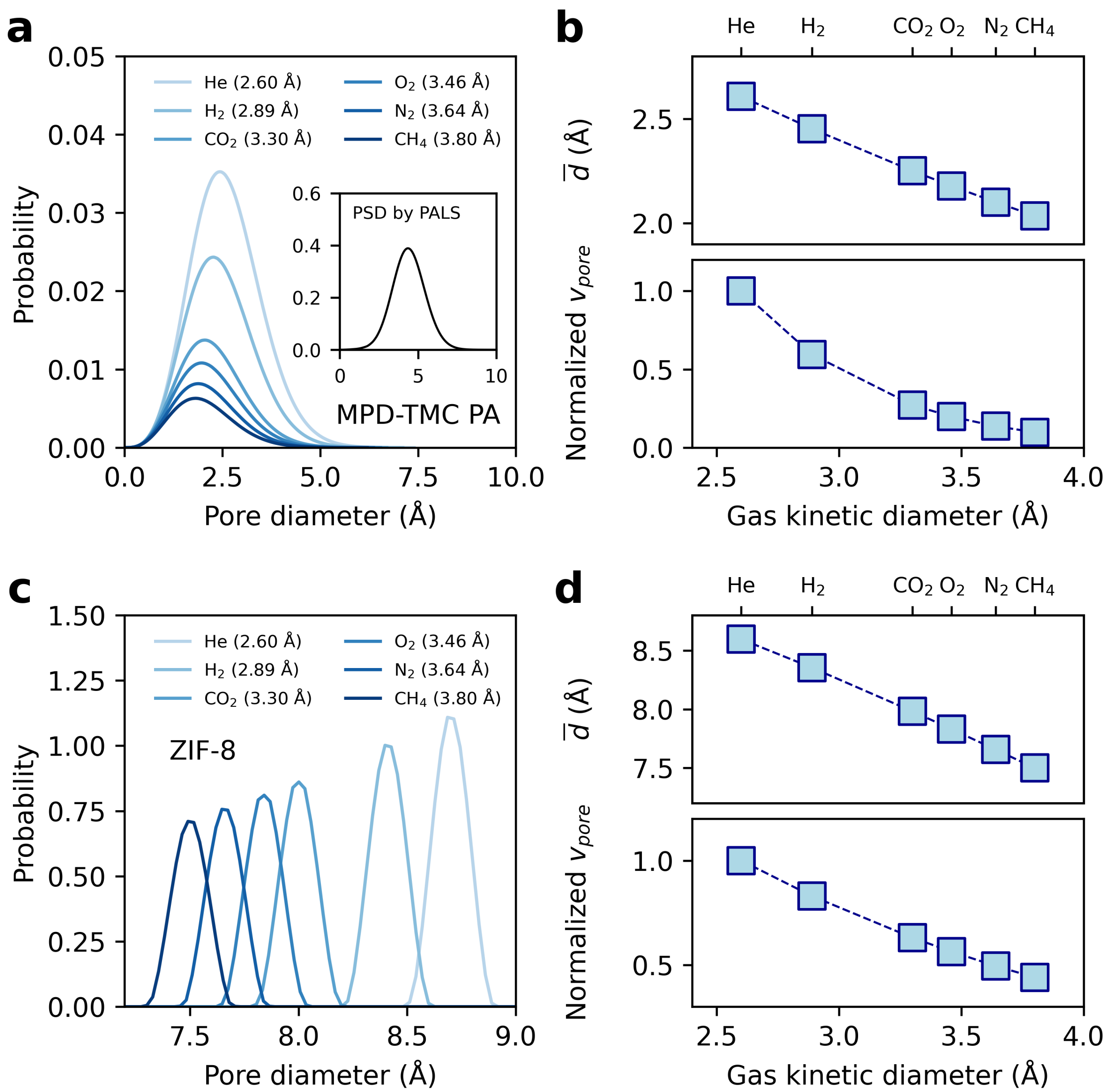

Fig. S7. The effective pore felt by gases with different kinetic diameter. a The pore size distribution of MPD-TMC PA for gases with different kinetic diameters calculated by Eq. 15. Inset: the pore size distribution of MPD-TMC PA obtained by PALS[5]. **b** The effective mean pore diameter and pore volume in MPD-TMC PA for gases with different kinetic diameters. **c**, **d** Same as **a** and **b**, respectively, but for ZIF-8. The pore size distribution of ZIF-8 was calculated using Zeo++[6]. Source data are provided as a Source Data file.

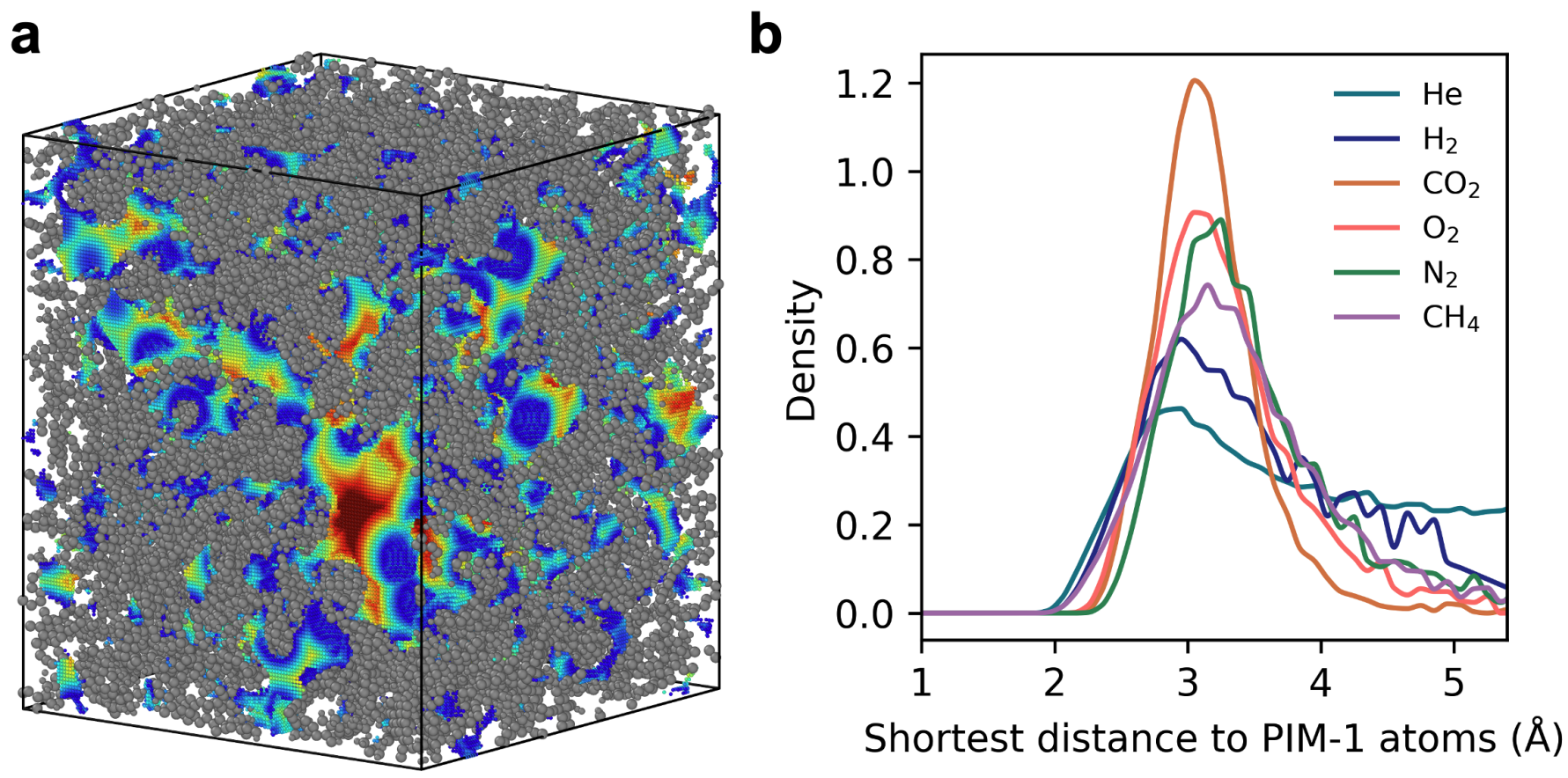

Fig. S8. Gas density distribution in PIM-1 pores. a Spatial distribution of pore regions in PIM-1. The blue regions indicate pore spaces close to the PIM-1 atoms, while the red regions correspond to pores farther away. Grey spheres represent PIM-1 atoms. **b** Gas density profiles as a function of distance from PIM-1 atoms, showing that CO_2 molecules preferentially occupy regions closer to the PIM-1 surface than other gases. Source data are provided as a Source Data file.

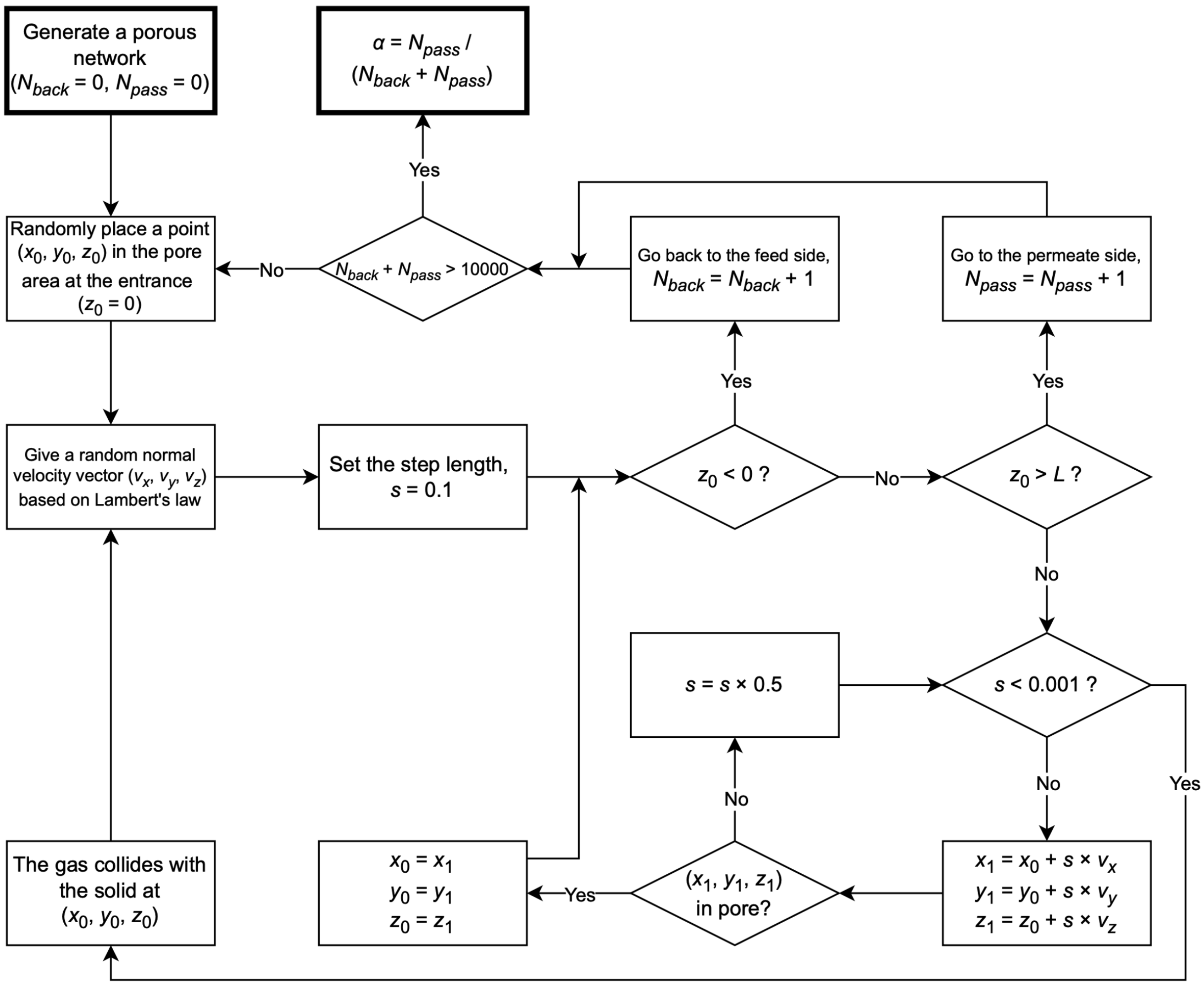

Fig. S9. Flow chart of MC simulations.

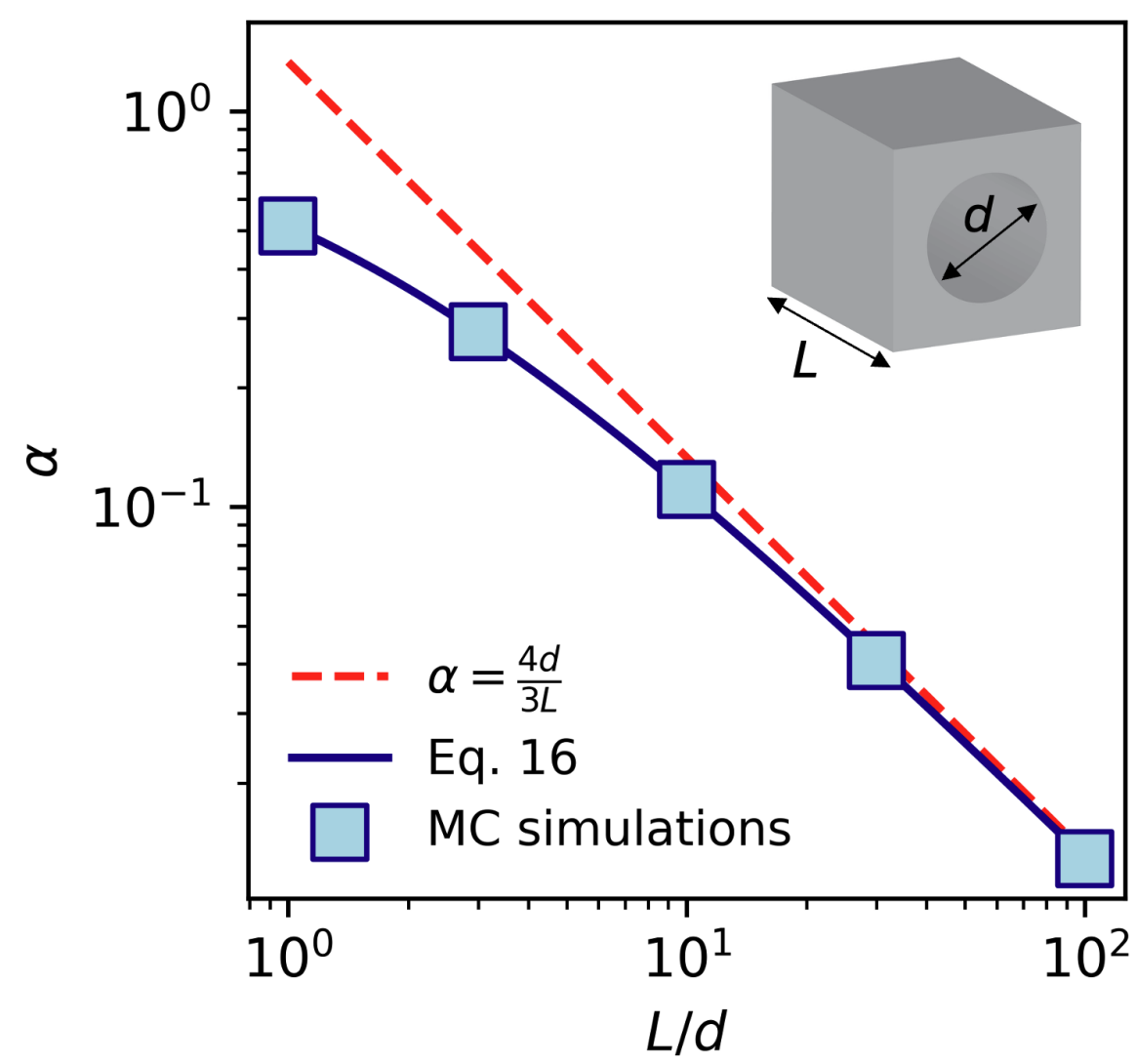

Fig. S10. Dependence of transmission coefficient (α) on the ratio L/d for straight circular channels. The square symbols denote Monte Carlo simulation results. The blue solid line represents Eq. (17), while the red dashed line shows the analytical prediction of the well-known Knudsen equation, $\alpha = 4d / 3L$. Inset: schematic illustration of the channel geometry with length L and diameter d. Source data are provided as a Source Data file.

References

1. Holt, J. K. *et al.* Fast Mass Transport Through Sub-2-Nanometer Carbon Nanotubes. *Science* **312**, 1034–1037 (2006).
2. Keerthi, A. *et al.* Ballistic molecular transport through two-dimensional channels. *Nature* **558**, 420–424 (2018).
3. Porodnov, B. T., Suetin, P. E., Borisov, S. F. & Akinshin, V. D. Experimental investigation of rarefied gas flow in different channels. *J. Fluid Mech.* **64**, 417–438 (1974).
4. Qian, J., Wu, H. & Wang, F. A generalized Knudsen theory for gas transport with specular and diffuse reflections. *Nat. Commun.* **14**, 7386 (2023).
5. Shen, L. *et al.* Polyamide-based membranes with structural homogeneity for ultrafast molecular sieving. *Nat. Commun.* **13**, 500 (2022).
6. Willems, T. F., Rycroft, C. H., Kazi, M., Meza, J. C. & Haranczyk, M. Algorithms and tools for high-throughput geometry-based analysis of crystalline porous materials. *Micropor. Mesopor. Mat.* **149**, 134–141 (2012).
