# Supplementary material for: A generalized Knudsen theory for gas transport in disordered porous materials
Source: Nat Commun. 2026 Jul 16;17:8719. doi: 10.1038/s41467-026-75656-8 (PMC13490521; doi:10.1038/s41467-026-75656-8)
Supplement: Supplementary file 2 [file 41467_2026_75656_MOESM2_ESM.pdf]

nature portfolio

Peer Review File

A generalized Knudsen theory for gas transport in disordered porous materials

Corresponding Author: Professor Menachem Elimelech

This file contains all reviewer reports in order by version, followed by all author rebuttals in order by version.

Version 0:

Reviewer comments:

Reviewer #1

(Remarks to the Author)
The authors make a series of approximations regarding the internal resistance in disordered materials and derive a simple result for the transmission probability and permeability of disordered materials. This results is validated against MC simulations. Because of the nature of the approximations and limitations of the model I do not believe it represents a sufficient advance to merit publication in Nature Communications. My reservations in this regard are as follows:

1. It is assumed that Knudsen diffusion prevails. In reality there can be a large surface slip and a momentum accommodation factor (or Maxwell reflection coefficient) that is significantly different from the value of unity applicable to Knudsen diffusion. This will depend on surface roughness. As an example a carbon nanotube has a very low reflection coefficient and a diffusivity value orders of magnitude larger than that for Knudsen diffusion. While a disordered medium may not have as smooth a surface, the diffusivity will in general be significantly different from the Knudsen value. This needs to be addressed in the model.

2. Another approximation is the neglect of fluid-solid interaction in the approach, as is the case for Knudsen diffusion, so that it is applicable only to weakly adsorbing gases such as helium or hydrogen. This is acknowledged in the manuscript, and is a significant limitation. The authors may note that this limitation of the Knudsen diffusivity has been examined at length in the literature (Chem. Eng. Sci. 2011, 66, 284-293). Even for weakly adsorbing gases there can be significant surface slip, so the previous reservation carries forward here as well.

3.While the authors tested their model against simulations, the re results are not convincing. For example, Figure 4g, which presents a comparison of MD and model-based permeabilities, shows significantly large error for ZIF8 or PA which is masked by the logarithmic scale used for permeability. One is not sure if the error is due to the model approximations regarding barriers or the deviation in surface slip (i.e. Maxwell coefficient different from unity).

4. The assumption of a barrier constriction ratio equal to the porosity does not seem to have any justification. Further, materials such as ZIF8 are not disordered and this concept should not be applicable.

As a minor point there is a typographical error in eqn. (3) in which 'l' should be 'L".

Reviewer #2

(Remarks to the Author)
This manuscript presents a generalized theoretical framework for Knudsen flow in random porous materials. By simplifying disordered porous networks into equivalent straight channels with internal barriers, the authors derive a simple permeability expression that depends only on porosity and mean pore size. The theory is validated against extensive MC simulations and NEMD simulations of real porous materials (PIM-1, polyamide, ZIF-8). The extension to gas selectivity in sub-nanometer pores by accounting for molecular size effects is also supported by experimental data for weakly adsorbing gases. The work offers a practical and accessible tool for predicting gas transport in nanoporous materials. Here are my specific comments
1. The manuscript successfully extends classical Knudsen theory from well-defined channels to disordered porous networks. The derived permeability equation is remarkably simple and depends only on two readily measurable parameters. This represents a significant advance for the gas separation and membrane communities, where existing models often rely

on hard-to-determine parameters such as tortuosity or fractal dimensions. The work is likely to be of broad interest.
2. The equivalent channel model with internal barriers is physically intuitive. The use of porosity as the constriction ratio and the assumption of $\chi=1$ (barrier spacing equals pore diameter) for isotropic random networks are well justified. However, the authors should clarify whether the assumption of $\chi=1$ holds for all isotropic networks, especially those with non-spherical pores or anisotropic connectivity. A brief discussion on the limits of this assumption would strengthen the manuscript.
3, The validation using 5000 random porous networks is comprehensive. The high R^2=0.986 is convincing. The authors also show that the theory performs better than existing tortuosity-based models. It would be useful to include a comparison with the classic Kozeny-Carman type models in the main text or Supplementary Information to further contextualize the improvement.
4. The NEMD simulations on PIM-1, ZIF-8, and polyamide provide strong support for the theory's applicability to real materials. The authors used elevated pressures (60-1200 bar) to ensure statistical sampling, which is acceptable. However, a comment on whether such high pressures could alter the pore structure or induce non-Knudsen effects (e.g., viscous flow) would be helpful. The authors note that previous work showed minimal deviation between 60 and 1200 bar, but this should be briefly reiterated here.
5. The treatment of molecular size effects by defining an effective pore size (d_eff = d – d_gas) is reasonable. The selectivity predictions for PIM-1, polyamide, and ZIF-8 show good agreement for weakly adsorbing gases. The authors correctly point out that the theory fails for strongly adsorbing gases like CO_2 due to surface adsorption and condensation. This limitation is clearly stated and is acceptable.
6. One of the key claims is that mean chord length and porosity from 2D cross-sections are statistically equivalent to 3D values for random porous media. This is critical for practical application. The authors should provide a brief justification or cite established stereological principles to support this claim.

Reviewer #3

(Remarks to the Author)
Here, the authors developed a generalized theoretical framework to describe Knudsen flow in disordered porous materials. Specifically, they developed a relationship between permeability, mean pore size, and porosity. They validated their theory against MC simulations with 5000 random porous networks. Furthermore, they performed NEMD simulations to demonstrate the applicability of their theory to realistic membranes such as polyamide. Deriving a relationship for permeability in random porous materials is almost impossible due to the complex channel geometry. However, the authors present a neat solution by simplifying the channels as cylindrical channels with internal barriers, allowing permeability (or transmission probability) to be expressed analytically. This is an elegant engineering approach, and given that the authors validated their theoretical framework, I recommend the manuscript for publication. I have the following comments and suggestions to further improve the manuscript:
-Currently, transport through the nanoporous material is modeled as a series of barriers in a 1D channel. What happens if a barrier is infinitely high (e.g., representing a dead end inside the porous materials)? Should the model instead be constructed based on multiple channels in parallel?
- It is not clear how structural features such as pore connectivity are captured in this formulation. For example, connectivity can strongly influence percolation pathways and the presence of dead-end pores. The authors may want to comment on how connectivity is implicitly reflected in parameters such as porosity or barriers.

Version 1:

Reviewer comments:

Reviewer #1

(Remarks to the Author)
The authors have largely addressed this reviewer's comments appropriately. Nevertheless, the usefulness of the theory remains tenuous. This is because it is applicable to only weakly adsorbing gases, and at normal conditions only helium and for sufficiently large pore sizes, hydrogen, will satisfy this requirement. For most important gases such as methane or carbon dioxide unrealistically high temperatures will be required before the theory becomes applicable.

A further weakness is that any disordered material will have a pore size distribution, which requires three parameters to describe it. These are the porosity, modal pore size, and standard deviation. The authors characterize a random medium by only the porosity and a single pore radius. How is this able to capture materials with a wide pore size distribution, and the effect of width of the pore size distribution on the permeability?

While I am not recommending that the manuscript be declined, I suggest the authors address these questions and build a stronger case for the importance and use of the approach. Otherwise, I suspect it is unlikely to achieve its intended impact.

Reviewer #2

(Remarks to the Author)
The authors have thoroughly addressed all my previous concerns with additional simulations, clarifications, and revised text.

The revised manuscript is now suitable for publication in Nature Communications. I recommend acceptance.

Reviewer #3

(Remarks to the Author)
The authors appropriately addressed my comments. Therefore, I recommend the publication of the manuscript in its current form.

Version 2:

Reviewer comments:

Reviewer #1

(Remarks to the Author)
The authors have addressed my comments and the manuscript may be accepted.

Open Access This Peer Review File is licensed under a Creative Commons Attribution 4.0 International License, which permits use, sharing, adaptation, distribution and reproduction in any medium or format, as long as you give appropriate credit to the original author(s) and the source, provide a link to the Creative Commons license, and indicate if changes were made.
In cases where reviewers are anonymous, credit should be given to 'Anonymous Referee' and the source.
The images or other third party material in this Peer Review File are included in the article's Creative Commons license, unless indicated otherwise in a credit line to the material. If material is not included in the article's Creative Commons license and your intended use is not permitted by statutory regulation or exceeds the permitted use, you will need to obtain permission directly from the copyright holder.
To view a copy of this license, visit https://creativecommons.org/licenses/by/4.0/

Point-by-point response to the reviewers' comments

Reviewer #1 (Remarks to the Author):

Before responding to the specific comments of the reviewers, we would like to highlight the key revisions made in response to all reviewers:

(1) ***Validation of Gas-Solid Interaction Assumptions***: To address concerns regarding the reflection mode and surface adsorption, we performed additional Monte Carlo and nonequilibrium molecular dynamics (NEMD) simulations. The results confirm that for weakly adsorbing gases in atomically rough, disordered porous networks, the assumptions of fully diffuse reflection and negligible surface adsorption are physically justified (added Text S1 and Figs. S5–S6).

(2) ***Confirmation of the Knudsen-Dominated Regime***: We introduced a collision-based analysis directly from the NEMD trajectories to quantify the ratio of gas-wall to gas-gas collisions. This confirms Knudsen transport dominates in our simulations (added Text S2).

(3) ***Clarification of Model Simplifications and Advantages***: We further explained how our model simplifies complex disordered porous networks into a channel with internal barriers (added Fig. S1). Additionally, we discussed the advantages of our framework over conventional tortuosity-based models.

The authors make a series of approximations regarding the internal resistance in disordered materials and derive a simple result for the transmission probability and permeability of disordered materials. This results is validated against MC simulations. Because of the nature of the approximations and limitations of the model I do not believe it represents a sufficient advance to merit publication in Nature Communications. My reservations in this regard are as follows:

Response: We sincerely thank the reviewer for the thoughtful evaluation of our manuscript. Regarding the concern about the significance of this advance, we respectfully emphasize that directly linking complex microscopic pore structures to macroscopic gas permeability in disordered media has conventionally relied on complex and empirical parameters or computationally simulations, both of which are impractical. In this work, we propose treating the porous network as a series of continuously stacked layers and analyze the gas transport resistance between adjacent layers. Compared to previous models that characterize gas transport pathways through parameters, such as tortuosity and connectivity, our approach offers a theoretical innovation that eliminates the reliance on parameters that are difficult to measure and typically treated empirically. This strategy enables us to develop an elegant model that is both accurate and practical. The reviewer raised specific concerns regarding our assumption of diffuse reflection in gas-solid interactions and our proposed link between macroscopic porosity and microscopic molecular backscattering resistance. We have addressed each point in detail below.

1. It is assumed that Knudsen diffusion prevails. In reality there can be a large surface slip and a momentum accommodation factor (or Maxwell reflection coefficient) that is significantly different

from the value of unity applicable to Knudsen diffusion. This will depend on surface roughness. As an example a carbon nanotube has a very low reflection coefficient and a diffusivity value orders of magnitude larger than that for Knudsen diffusion. While a disordered medium may not have as smooth a surface, the diffusivity will in general be significantly different from the Knudsen value. This needs to be addressed in the model.

Response: We thank the Reviewer for this insightful comment. We fully agree that the Maxwell reflection coefficient plays an important role in gas transport through nanopores. This is particularly true in straight nanopores with atomically smooth walls, such as carbon nanotubes, graphene channels, and hBN channels, where specular reflection can lead to gas flux magnitudes larger than those predicted by Knudsen diffusion under the assumption of fully diffuse reflection [*Nature* 2018, 558 (7710), 420–424; *Science* 2006, 312 (5776), 1034–1037]. In the previous work, we have already developed the Knudsen theory incorporating the Maxwell reflection coefficient to describe the gas flux enhancement caused by specular reflection in straight channels, yielding a simple, accurate and physically meaningful equation [*Nat. Commun.* 2023, 14 (1), 7386].

However, we note that disordered porous media differ from these smooth and straight channels in two key aspects. First, unlike straight channels where specular reflection allows all molecules to travel to the permeate side, the structural barriers in porous networks redirect molecules back toward the feed side regardless of the reflection type. Therefore, flux dissipation in porous networks with low porosity is mainly caused by these barriers rather than diffuse reflection, making the enhancement by specular reflection less significant. Second, and more importantly, the walls of most disordered porous media are atomically rough, which brings the Maxwell reflection coefficient close to unity and satisfies the diffuse reflection assumption from classical Knudsen theory. For example, helium on glass, an amorphous material similar to PIM-1 and MPD-TMC PA, has a reported momentum accommodation factor of 0.935 [*J. Fluid Mech.* 1974, 64 (3), 417–438], which is numerically equivalent to the Maxwell reflection coefficient.

To further quantitatively assess the effect of the Maxwell reflection coefficient on gas transport in disordere porous network, we performed additional Monte Carlo simulations. Following the methods of Monte Carlo simulations described in this paper (Fig. 3c), we introduced the Maxwell reflection coefficient, f, where a molecule colliding with the wall undergoes diffuse reflection with probability f, and specular reflection with probability $1 - f$ (Fig. R1a). We selected porous networks generated by the same method as in Fig. 3a. For each of 800 generated porous structures, we calculated the gas transmission probability α at f = 0.0, 0.2, 0.4, 0.6, 0.8, and 1.0, and defined an enhancement factor as $\alpha(f)$ / $\alpha(f = 1)$ to quantify the increase in gas flux due to specular reflection, which causes surface slip.

The results show that a clear enhancement only occurs when f approaches 0 (Fig. R1b). Given that most common atomically rough surfaces have f values close to 1 [*J. Vac. Sci. Technol. A* 2008, 26 (4), 634–645], the assumption of purely diffuse, Knudsen-type reflection introduces an acceptable deviation of less than 10–20% for the systems studied here.

In the revised manuscript, we added Text S1 and Fig. S5 to discuss the influence of the Maxwell reflection coefficient.

Line 234: *"The current model assumes that gas molecules undergo fully diffuse reflection upon colliding with pore walls, which is the standard assumption of classical Knudsen theory. However, in atomically smooth straight channels like carbon nanotubes or graphene, specular reflection can lead to gas fluxes much higher than those predicted by classical Knudsen theory [Science 312, 1034–1037 (2006); Nature 558, 420–424 (2018)]. In previous work, specular reflection was considered to quantify this flux enhancement in straight channels [Nat. Commun. 14, 7386 (2023)]. For disordered porous media, however, internal structural barriers force molecules to backscatter regardless of the reflection mode. In addition, most materials typically exhibit atomic-scale roughness, which promotes diffuse gas reflection29. These factors substantially reduce flux enhancement associated with specular reflection. Therefore, the assumption of fully diffuse reflection is reasonable for such disordered porous systems (Supplementary Text S1 and Supplementary Fig. 5)."*

"Text S1. Effect of Maxwell reflection coefficient on gas transmission in disordered porous networks

The mode of gas reflection at the pore wall has a strong influence on gas transport, and is commonly described by the Maxwell reflection coefficient, f, where a gas molecule colliding with the wall undergoes diffuse reflection with probability f and specular reflection with probability $1 - f$. The derivation in this work assumes fully diffuse reflection ($f = 1$). Below we examine the deviation arising from applying the $f = 1$ assumption when $f < 1$.

In smooth channels such as carbon nanotubes, graphene channels, and hBN channels, specular reflection dominates (f approaching 0), producing gas fluxes much larger than those predicted by classical Knudsen diffusion[1,2]. However, disordered porous media differ from smooth straight channels in two ways. First, in straight channels, specular reflection allows molecules to maintain their forward momentum and travel toward the permeate side. In disordered porous networks, structural barriers and pore constrictions can redirect molecules back toward the feed side regardless of whether the gas–wall reflection is diffuse or specular. Therefore, the main source of flux dissipation in disordered porous networks with low porosity is the internal structural resistance, rather than diffuse gas–wall reflection alone. Second, most disordered porous materials have atomically rough internal surfaces. Such roughness brings the Maxwell reflection coefficient close to unity, making the fully diffuse reflection assumption reasonable. For example, helium on glass, an amorphous surface that is more representative of PIM-1 and MPD-TMC polyamide than atomically smooth wall, has a reported momentum accommodation factor of 0.935, which corresponds closely to f in the Maxwell reflection model.

To quantify the influence of f on transport in disordered porous networks, we performed additional Monte Carlo simulations using the porous networks generated by the same method as in Fig. 3a. The Maxwell reflection coefficient was introduced by assigning diffuse reflection with probability f and specular reflection with probability $1 - f$ upon each gas–solid collision. For each of 800 generated porous structures, we calculated the transmission probability α_{MC} at $f = 0, 0.2, 0.4, 0.6, 0.8$, and 1.0. The enhancement caused by specular reflection was quantified as $\alpha_{MC}(f) / \alpha_{MC}(f = 1)$.

The results show that significant enhancement appears only when f approaches 0, corresponding to nearly specular reflection. When f is close to unity, as expected for most atomically rough surfaces, the deviation from the purely diffuse Knudsen-type assumption is acceptable given the simplicity of the model."

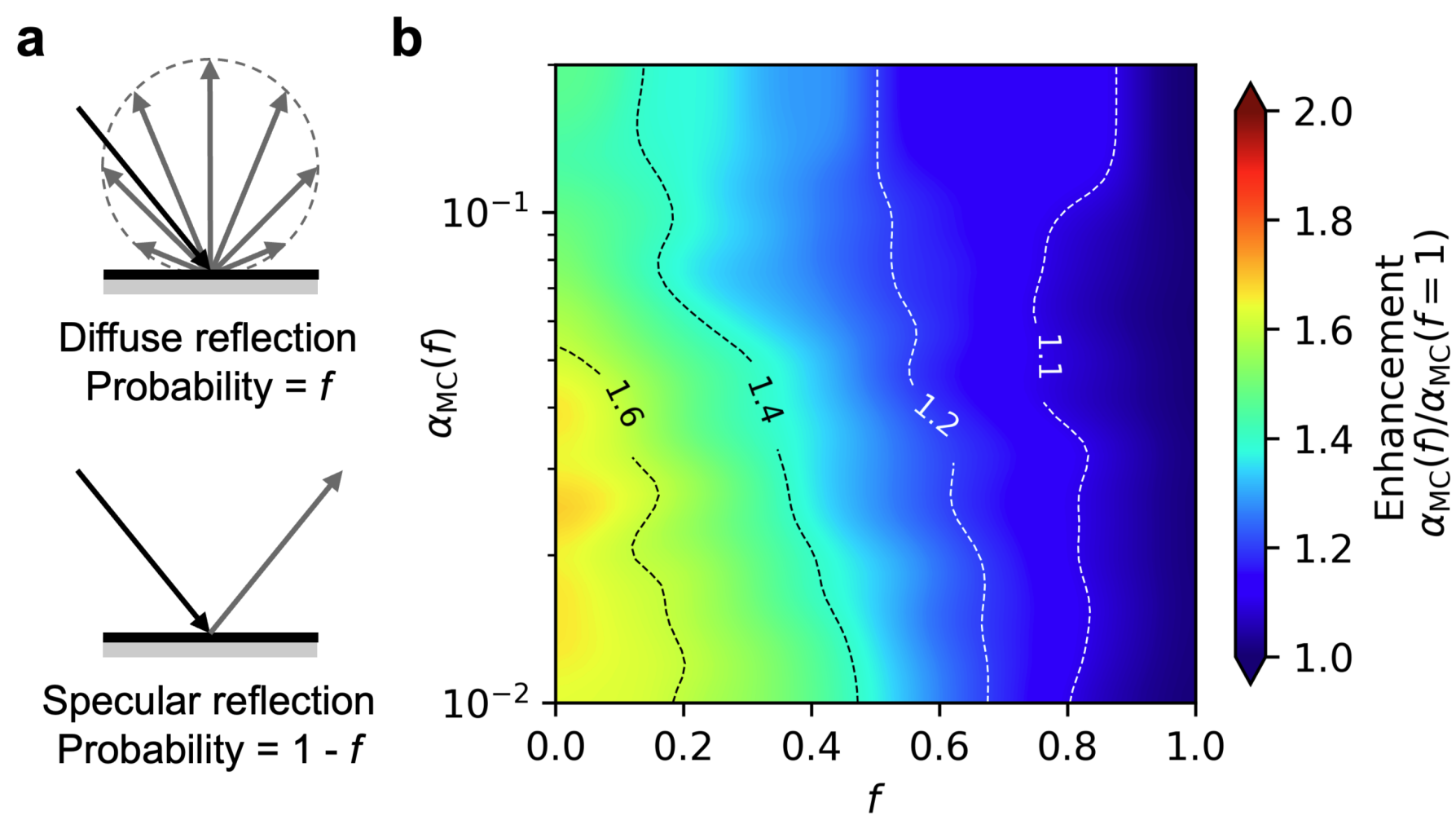

(Fig. R1) **Fig. S5. Effect of the Maxwell reflection coefficient on transmission probability enhancement in porous networks. a** Definition of the Maxwell reflection coefficient, *f*. When a gas molecule collides with a solid surface, it undergoes diffuse reflection with probability *f* and specular reflection with probability 1 − *f*. **b** Transmission probability enhancement, $\alpha_{MC}(f)$ / $\alpha_{MC}(f = 1)$, calculated from Monte Carlo simulations. Each data point represents the transmission probability $\alpha_{MC}(f)$ of one porous network at a given diffuse-reflection probability *f*. The enhancement is obtained by normalizing this value by $\alpha_{MC}(f = 1)$, calculated for the same porous network under fully diffuse reflection. The contour map was constructed from 4800 data points, based on 800 porous network structures with *L* = 200, each evaluated at *f* = 0, 0.2, 0.4, 0.6, 0.8, and 1.0. Dashed lines denote representative enhancement levels.

2. Another approximation is the neglect of fluid-solid interaction in the approach, as is the case for Knudsen diffusion, so that it is applicable only to weakly adsorbing gases such as helium or hydrogen. This is acknowledged in the manuscript, and is a significant limitation. The authors may note that this limitation of the Knudsen diffusivity has been examined at length in the literature (Chem. Eng. Sci. 2011, 66, 284-293). Even for weakly adsorbing gases there can be significant surface slip, so the previous reservation carries forward here as well.

Response: We appreciate the Reviewer's insightful comment. We fully agree that gas-solid interactions can significantly affect gas transport in nanopores, particularly for strongly adsorbing gases such as CO_2, for which Knudsen theory is known to break down. This limitation has been acknowledged in the manuscript. Here we provide additional evidence to demonstrate that, for weakly adsorbing gases like helium, this effect is negligible and the Knudsen diffusion assumption remains valid.

We performed an additional NEMD simulation of helium transport through a 2 nm slit channel constructed from MPD-TMC polyamide membranes under 1 atm pressure difference, following the same simulation protocol and flux calculation procedure as described in the Methods section. We calculated the theoretical flux using the Knudsen model that accounts for specular reflection [Nat. Commun. 2023, 14 (1), 7386]:

$$J = \frac{\alpha_K}{f + \alpha_K - f\alpha_K} \frac{\Delta PA}{\sqrt{2\pi RTM}}$$

where f is the Maxwell reflection coefficient, α_K is the transmission probability when f = 1, calculated using the formula derived by Berman [*J. Appl. Phys.* 1965, 36 (10), 3356–3356], ΔP is the pressure difference across the membrane, A is the cross-sectional area, R is the gas constant, T is the temperature, and M is the molar mass of the gas molecule. We found that when f = 1, the theoretical flux underestimates the simulated value by approximately 5.8%. When f = 0.9, however, the theoretical flux agrees well with the MD results (Fig. R2). This further confirms that the Maxwell reflection coefficient of helium on MPD-TMC polyamide is close to 0.9, and also suggests that assuming f = 1 introduces only a small underestimation of the flux for systems where f = 0.9 in this case.

To understand the physical origin of this agreement, we further analyzed the helium density distribution across the channel (Fig. R2). No pronounced density peak was observed near the polyamide wall, confirming that helium molecules do not adsorb appreciably on the surface. This provides a molecular-level explanation for why the Knudsen assumption holds well in this system.

We note that this finding is consistent with what was reported in the literature cited by the Reviewer (Chem. Eng. Sci. 2011, 66, 284–293), where deviations from Knudsen diffusion arise primarily from surface adsorption. Since helium adsorption on polyamide walls is negligible, such deviations are not expected in our system.

We therefore conclude that, for weakly adsorbing gases, neglecting fluid-solid interactions is a reasonable simplification in this work. In the revised manuscript, we have added a discussion on the validity of neglecting gas adsorption and surface diffusion for weakly adsorbing gases (helium), including the applicability of the Knudsen model, along with Supplementary Fig. SX.

Line 261: *" Our theory describes Knudsen transport, in which gas molecules undergo diffuse reflections upon collision with pore walls. Helium was selected for the simulations because gases with stronger intermolecular interactions can exhibit significant adsorption and surface diffusion within the membrane, thereby obscuring direct comparisons with the Knudsen-based framework [Chem. Eng. Sci. 66, 284–293 (2011)]. By contrast, helium interacts only weakly with membrane atoms and exhibits negligible surface adsorption (Fig. 4b). This assumption was further validated through simulations of helium transport in a well-defined slit pore formed by MPD-TMC PA membranes. In this system, the simulated gas flux agreed closely with the prediction of Knudsen theory and did not exhibit appreciable surface slip or localized density enhancements near the pore walls (Supplementary Fig. 6)."*

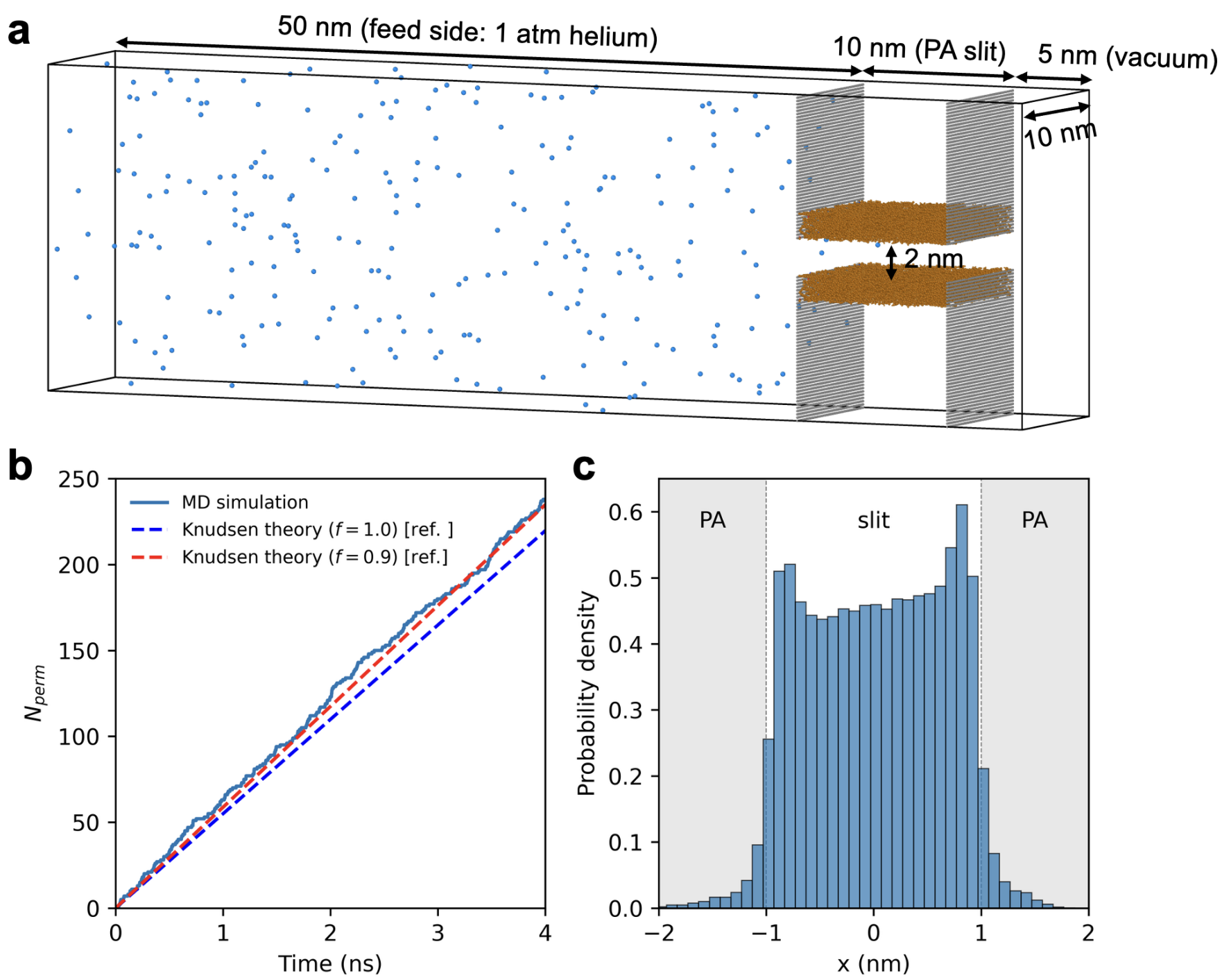

(Fig. R2) Fig. S6. Helium permeation through a polyamide slit and validation of the Knudsen model. a Setup for nonequilibrium molecular dynamics simulations of helium gas permeation through a slit 2 nm wide and 10 nm long, formed by two MPD-TMC PA sheets. Monatomic carbon sheets separate the feed side from the vacuum permeate side, ensuring that helium gas only passes through the PA slit. Helium atoms are colored blue and visually enlarged, PA membranes are dark orange, and carbon sheets are black. b The number of helium atoms permeating through the PA slit over time, compared with the Knudsen theory assuming fully diffuse reflection ($f = 1$) and partial diffuse reflection ($f = 0.9$), where gas molecules colliding with the wall have a f probability of diffuse reflection and a $1 - f$ probability of specular reflection. c The density profile of helium gas in the slit along the normal direction of the PA membrane.

3.While the authors tested their model against simulations, the re results are not convincing. For example, Figure 4g, which presents a comparison of MD and model-based permeabilities, shows significantly large error for ZIF8 or PA which is masked by the logarithmic scale used for permeability. One is not sure if the error is due to the model approximations regarding barriers or the deviation in surface slip (i.e. Maxwell coefficient different from unity).

Response: We sincerely thank the reviewer for the careful evaluation of Figure 4g and for raising this insightful question regarding the sources of deviation. We would like to clarify that the logarithmic scale was used for permeability because of the wide range of values across the tested systems, which span orders of magnitude from approximately 5 Barrer to 30,000 Barrer. To provide a clearer and more direct comparison between theoretical and simulated values, we list the absolute permeability values (in Barrer) and the corresponding relative errors for the three representative systems below:

- He-PIM1 (Theory: 9581, MD simulation: 11235, Relative Error: 17.26%);
- He-ZIF8 (Theory: 30167, MD simulation: 22375, Relative Error: 25.82%);
- He-PA (Theory: 3.91, MD simulation: 5.16, Relative Error: 31.86%).

As the reviewer pointed out, the observed deviations may partly stem from some assumptions in our theoretical model. Specifically, our theoretical framework assumes a disordered porous network. Applying this assumption to ZIF-8, which is an ordered crystalline framework, introduces an inherent structural mismatch that likely contributes to the observed discrepancy. For PA, the simulated permeability exceeds the theoretical prediction, which is consistent with the reviewer's suggestion that He transport in PA may not follow purely diffuse reflection. Based on our calculations of He flow through a 2 nm slit formed by PA walls (Fig. R2), the Maxwell reflection coefficient for He on PA surfaces is estimated to be around 0.9. A coefficient below unity introduces a partial specular reflection component that enhances flux beyond the purely diffuse limit, leading to higher simulated permeabilities than the theoretical prediction.

Beyond these factors, we would like to note several additional reasons why the model cannot perfectly reproduce gas transport in real porous membranes. For PA, the internal pore dimensions are on the order of a few angstroms, at which scale thermal fluctuations of the membrane atoms cause dynamic variations in pore size, rather than the static pore geometry assumed in our theoretical framework. Gas transport through such dynamically evolving pores may yield higher effective permeability than the static model predicts. In addition, our definition of solid and void regions relies on van der Waals radii, which is an idealization. In reality, the effective contact distance between two colliding atoms decreases with increasing kinetic energy, meaning the accessible pore volume is subtly dependent on gas kinetic energy, which follows a statistical distribution rather than a single fixed value.

These factors collectively make a perfectly accurate description of gas transport in such membranes inherently challenging. In membrane-based gas separation, permeability differences between materials typically span multiple orders of magnitude, and the practically relevant question is whether a model correctly captures the relative ranking and order-of-magnitude behavior across diverse materials with ease. Our model achieves this across approximately four orders of magnitude (~5 to ~30,000 Barrer), correctly predicting both the ranking and the magnitude for all tested systems, with a relative error below 32%. We therefore consider this level of agreement to be acceptable and sufficient for practical purposes such as materials screening and design.

In the revised manuscript, we have added a discussion of potential sources of deviation.

Line X: *"Third, when pore sizes approach molecular dimensions, thermal motions of membrane atoms create dynamic pores that can influence gas transport. Although our results demonstrate good predictive capability for such membranes, the present framework does not explicitly account for these dynamic pore fluctuations. Fourth, when the porous medium contains atomically smooth walls, the effect of specular reflection on gas flux should be considered explicitly. Future work will integrate adsorption effects, pore dynamics, and reflection type to further refine this framework and extend its applicability."*

4a. The assumption of a barrier constriction ratio equal to the porosity does not seem to have any justification ...

Response: We thank the reviewer for pointing out the need for further clarification on this assumption. The barrier constriction ratio in our equivalent 1D channel model is not a geometric approximation, but rather a statistical representation of the back-scattering probability inherent in 3D random porous networks. In a completely random and isotropic porous material, the pore distribution in any given 2D cross-section is statistically independent of the adjacent cross-section at a distance characteristic of the pore length (Fig. R3). When a gas molecule travels from one pore layer to the next, the probability of encountering an open void space in the forward direction is strictly governed by the macroscopic porosity of the material.

To translate this 3D transport resistance into our simplified 1D barrier-channel model, we intend the model to represent this specific probability of forward transmission versus back-scattering. In the 1D model, the probability of a gas molecule passing through the internal barrier without reflecting back is directly determined by the ratio of the barrier opening area to the total channel cross-sectional area, defined as the constriction ratio. Therefore, setting the barrier constriction ratio equal to the porosity ensures that the 1D model mathematically preserves the back-scattering resistance that a molecule experiences when crossing independent pore layers in the actual random network.

In the revised manuscript, we have added Fig. S1 to help readers better understand our equivalent strategy.

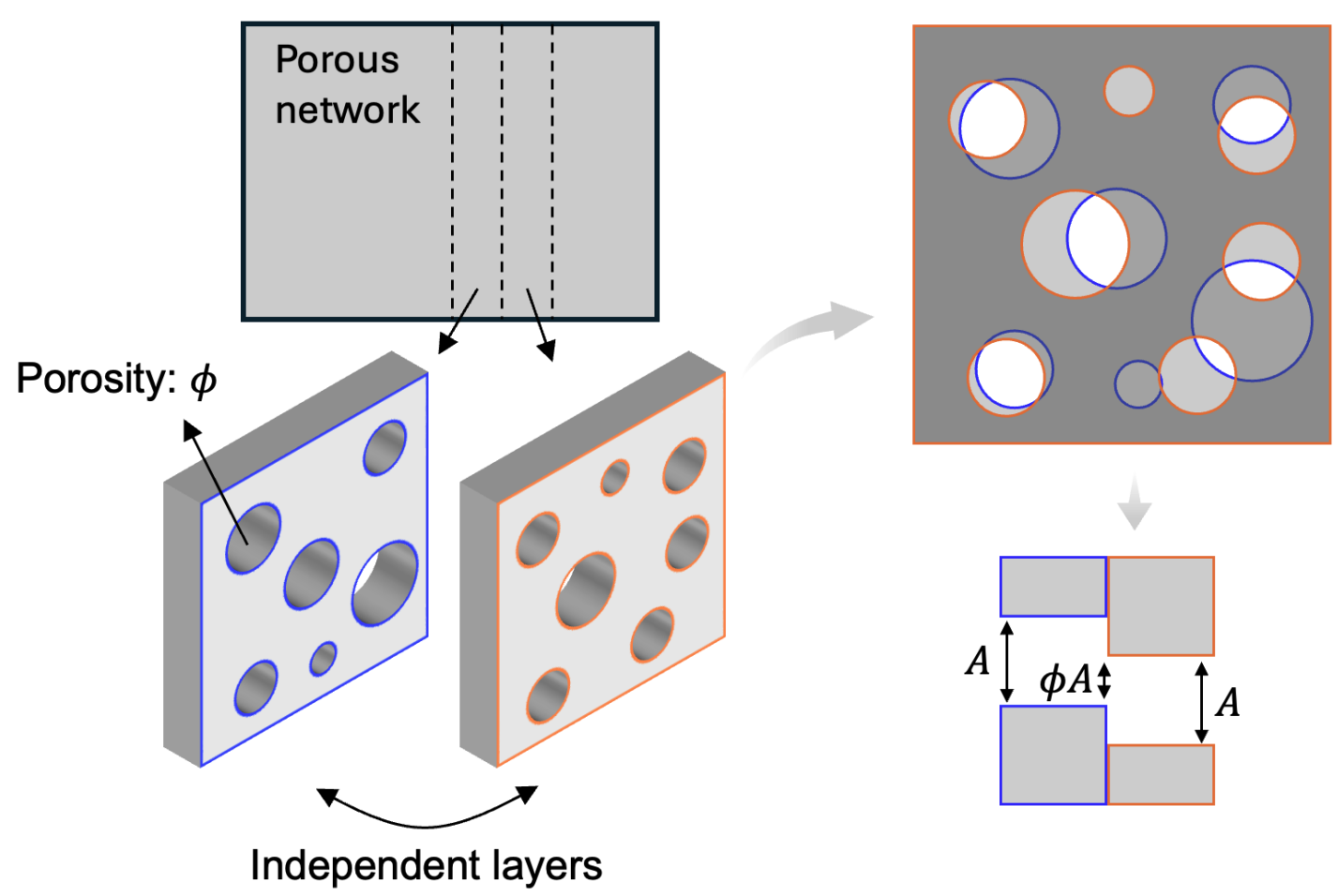

(Fig. R3) Fig. S1. Equivalence of the barrier constriction ratio of the 1D channel model as the porous network porosity.

4b ... Further, materials such as ZIF8 are not disordered and this concept should not be applicable.

Response: We thank the reviewer for pointing this out. We fully agree that ZIF-8 is a highly ordered crystalline material and that the derivation of our present framework is strictly based on random porous media.

Our intention in including ZIF-8 was not to classify it as a disordered network. The central transport mechanism captured by our model relies on geometric obstruction and back-scattering. Because the characteristic cavity-window structure of MOFs like ZIF-8 inherently creates distinct internal geometric barriers, we selected it as an exploratory test case. This allows us to examine how the physical assumptions of our barrier model behave in an ordered system featuring repeated pore constrictions.

We agree that the applicability of the framework to ordered materials must be discussed carefully to avoid misleading readers. Accordingly, we have clarified our rationale for including ZIF-8 in the revised manuscript.In the revised manuscript, we clarified the rationale for adding ZIF-8:

Line 255: *" Three representative membrane materials—PIM-1 (Fig. 4a), ZIF-8 (Fig. 4c), and MPD-TMC polyamide (PA) (Fig. 4d)—were selected to span a broad range of porosities and structural order. Although random porous networks constitute the primary target of our theoretical framework, ZIF-8 was included as an additional exploratory test system. Its characteristic cavity–window architecture introduces well-defined internal geometric barriers, providing an opportunity to evaluate the physical assumptions underlying the barrier model in a highly ordered porous material."*

As a minor point there is a typographical error in eqn. (3) in which 'l' should be 'L".

Response: Thank the Reviewer for this careful read. We have corrected this typographical error.

Reviewer #2 (Remarks to the Author):

Before responding to the specific comments of the reviewers, we would like to highlight the key revisions made in response to all reviewers:

(1) ***Validation of Gas-Solid Interaction Assumptions***: To address concerns regarding the reflection mode and surface adsorption, we performed additional Monte Carlo and nonequilibrium molecular dynamics (NEMD) simulations. The results confirm that for weakly adsorbing gases in atomically rough, disordered porous networks, the assumptions of fully diffuse reflection and negligible surface adsorption are physically justified (added Text S1 and Figs. S5–S6).

(2) ***Confirmation of the Knudsen-Dominated Regime***: We introduced a collision-based analysis directly from the NEMD trajectories to quantify the ratio of gas-wall to gas-gas collisions. This confirms Knudsen transport dominates in our simulations (added Text S2).

(3) ***Clarification of Model Simplifications and Advantages***: We further explained how our model simplifies complex disordered porous networks into a channel with internal barriers (added Fig. S1). Additionally, we discussed the advantages of our framework over conventional tortuosity-based models.

This manuscript presents a generalized theoretical framework for Knudsen flow in random porous materials. By simplifying disordered porous networks into equivalent straight channels with internal barriers, the authors derive a simple permeability expression that depends only on porosity and mean pore size. The theory is validated against extensive MC simulations and NEMD simulations of real porous materials (PIM-1, polyamide, ZIF-8). The extension to gas selectivity in sub-nanometer pores by accounting for molecular size effects is also supported by experimental data for weakly adsorbing gases. The work offers a practical and accessible tool for predicting gas transport in nanoporous materials. Here are my specific comments

Response: We sincerely thank the reviewer for the careful reading of our manuscript and the positive evaluation of our work. Below, we provide a detailed, point-by-point response to specific comments.

1. The manuscript successfully extends classical Knudsen theory from well-defined channels to disordered porous networks. The derived permeability equation is remarkably simple and depends only on two readily measurable parameters. This represents a significant advance for the gas separation and membrane communities, where existing models often rely on hard-to-determine parameters such as tortuosity or fractal dimensions. The work is likely to be of broad interest.

Response: We sincerely thank the reviewer for the positive evaluation and for recognizing the practical utility and simplicity of our derived permeability model in disordered porous networks.

2. The equivalent channel model with internal barriers is physically intuitive. The use of porosity as the constriction ratio and the assumption of $\chi=1$ (barrier spacing equals pore diameter) for isotropic random networks are well justified. However, the authors should clarify whether the

assumption of χ=1 holds for all isotropic networks, especially those with non-spherical pores or anisotropic connectivity. A brief discussion on the limits of this assumption would strengthen the manuscript.

Response: We thank the reviewer for this insightful comment. In our model, χ is defined as the ratio of the barrier spacing to the characteristic channel diameter. For a homogeneous and statistically isotropic random network, there is no preferred structural length scale along the transport direction, so the characteristic distance between successive barriers is statistically expected to be comparable to the characteristic pore size, leading to χ = 1. This assumption does not require each pore to be spherical. For non-spherical pores with random orientations, the shape anisotropy is statistically averaged over the network. However, χ = 1 does not hold for structures with preferentially oriented pores, or anisotropic connectivity. In such cases, the spacing between successive constrictions along the transport direction may differ from the channel diameter, and χ should be treated as a separate structural parameter. We have revised the manuscript to clarify the limit of the χ = 1 assumption.

Line 448: *" Second, our framework and the simplification of χ = 1 assume fully random, isotropic porous networks. For highly ordered materials such as aligned one-dimensional pores, classical Knudsen theory is sufficient. For anisotropic networks with randomly oriented, non-spherical pores, χ should be treated as an independent structural parameter."*

3. The validation using 5000 random porous networks is comprehensive. The high R^2=0.986 is convincing. The authors also show that the theory performs better than existing tortuosity-based models. It would be useful to include a comparison with the classic Kozeny-Carman type models in the main text or Supplementary Information to further contextualize the improvement.

Response: We thank the reviewer for suggesting this comparison to further contextualize our work. However, the classic Kozeny-Carman model and our proposed framework operate in fundamentally different physical regimes. The Kozeny-Carman equation describes viscous continuum flow governed by fluid-fluid collisions. In contrast, our model addresses the purely ballistic Knudsen transport regime, which is governed entirely by gas-wall collisions. Because of this distinct mechanistic difference, directly applying the Kozeny-Carman equation to our Knudsen flow systems for a numerical comparison is not physically applicable.

4. The NEMD simulations on PIM-1, ZIF-8, and polyamide provide strong support for the theory's applicability to real materials. The authors used elevated pressures (60-1200 bar) to ensure statistical sampling, which is acceptable. However, a comment on whether such high pressures could alter the pore structure or induce non-Knudsen effects (e.g., viscous flow) would be helpful. The authors note that previous work showed minimal deviation between 60 and 1200 bar, but this should be briefly reiterated here.

Response: We thank the reviewer for raising this important point. The materials considered here, including PIM-1, ZIF-8, and crosslinked MPD-TMC PA, are relatively rigid nanoporous materials. Within the pressure range used in our NEMD simulations, no significant pressure-induced pore

deformation was observed. More importantly, the theoretical predictions were not calculated based on the pore structure at room pressure. Instead, the porosity and mean pore size used in the theoretical prediction were obtained from the corresponding simulated structure under each pressure condition. Therefore, any small pressure-induced structural change, if present, was already included in the theoretical calculation. This ensures a consistent comparison between the NEMD results and the theoretical predictions. We have added a brief clarification on this point in the revised manuscript.

Line 297: *"For each membrane, the effective porosity and mean pore size were calculated from the membrane configurations obtained under the corresponding NEMD pressure conditions ..."*

To examine whether the elevated pressures induce non-Knudsen transport, we evaluated the relative frequency of gas-wall and gas-gas collisions directly from the NEMD trajectories. In disordered porous materials, estimating a conventional Knudsen number from a single pore size is not straightforward because the pore geometry is highly heterogeneous. Therefore, we used a collision-based analysis that directly reflects the physical origin of Knudsen transport, namely whether gas-wall collisions dominate over gas-gas collisions. Specifically, a collision event was identified when the van der Waals interaction between two atoms entered the repulsive regime, corresponding to a positive van der Waals pair energy. We calculated the probability of He-solid repulsive contacts and compared it with the probability of He-He repulsive contacts. The ratio between these two probabilities provides a direct measure of the relative importance of gas-wall and gas-gas collisions.

For PIM-1 at a feed-side pressure of 60 bar, the gas-wall collision probability is 23.5 times higher than the gas-gas collision probability. For ZIF-8 at 60 bar, this ratio is 13.8. For polyamide at 1200 bar, the ratio is still 6.0. These results show that gas-wall collisions remain dominant even under the elevated pressures used in our simulations. Therefore, the transport remains in the Knudsen-dominated regime rather than transitioning to viscous-flow behavior. We have added this analysis and clarification to the revised manuscript.

Line 281: "Under these elevated pressure conditions, helium transport in these membranes remains in the Knudsen-dominated regime (Supplementary Text S2)."

Text S2: "Collision-based assessment of the Knudsen-dominated regime

To examine whether the elevated pressures used in the NEMD simulations could induce non-Knudsen transport, we evaluated the relative importance of gas-wall and gas-gas collisions directly from the NEMD trajectories. For disordered porous materials, estimating a conventional Knudsen number using a single characteristic pore size is not straightforward because of the heterogeneous pore geometry. Therefore, we used a collision-based analysis to directly assess whether gas-wall collisions remain dominant over gas-gas collisions.

Specifically, membrane configurations were extracted from the NEMD simulations. For each helium atom located inside the membrane, we calculated its pairwise van der Waals interaction energy with other helium atoms and with membrane atoms. A collision was identified when the pairwise van der Waals interaction entered the repulsive regime, corresponding to a positive van der Waals pair energy. Thus, a He-He collision was counted when a helium atom had a positive van der Waals pair energy with another helium atom, while a He-solid collision was counted when it had a positive

van der Waals pair energy with any membrane atom. The analysis was performed only for helium atoms located in the membrane interior, excluding those within 1 nm of the membrane boundaries.

We then calculated, for each helium atom, the probability of experiencing He-solid repulsive contacts and the probability of experiencing He-He repulsive contacts. The ratio of these two probabilities was used to quantify the relative importance of gas-wall and gas-gas collisions. For PIM-1 at a feed-side pressure of 60 bar, the gas-wall collision probability was 23.5 times higher than the gas-gas collision probability. For ZIF-8 at 60 bar, this ratio was 13.8. For MPD-TMC PA at 1200 bar, the ratio was still 6.0. These results show that gas-wall collisions remain dominant in all three membranes, even under elevated pressures. Therefore, the simulated transport remains in the Knudsen-dominated regime rather than transitioning to viscous-flow behavior."

5. The treatment of molecular size effects by defining an effective pore size (d_eff = d − d_gas) is reasonable. The selectivity predictions for PIM-1, polyamide, and ZIF-8 show good agreement for weakly adsorbing gases. The authors correctly point out that the theory fails for strongly adsorbing gases like CO_2 due to surface adsorption and condensation. This limitation is clearly stated and is acceptable.

Response: Thank the Reviewer for the positive feedback.

6. One of the key claims is that mean chord length and porosity from 2D cross-sections are statistically equivalent to 3D values for random porous media. This is critical for practical application. The authors should provide a brief justification or cite established stereological principles to support this claim.

Response: Thank the Reviewer for pointing this out. We apologize that the original manuscript did not cite established stereological principles to support this claim, which may have caused confusion. The statistical equivalence of porosity and mean chord length measured from 2D cross-sections and their corresponding 3D values in random porous media is well-established in stereology. Underwood explicitly discussed this principle in his article "Stereology, or the Quantitative Evaluation of Microstructures" (*Journal of Microscopy* 1969, 89, 161–180). In the revised manuscript, we have added this reference to support our statement.

Line 181: *" Crucially, for random porous media, established stereological principles show that the 3D mean chord length and porosity are statistically equivalent to those measured from 2D cross-sections [Journal of Microscopy 1969, 89 (2), 161–180]."*

Reviewer #3 (Remarks to the Author):

Before responding to the specific comments of the reviewers, we would like to highlight the key revisions made in response to all reviewers:

(1) ***Validation of Gas-Solid Interaction Assumptions***: To address concerns regarding the reflection mode and surface adsorption, we performed additional Monte Carlo and nonequilibrium molecular dynamics (NEMD) simulations. The results confirm that for weakly adsorbing gases in atomically rough, disordered porous networks, the assumptions of fully diffuse reflection and negligible surface adsorption are physically justified (added Text S1 and Figs. S5–S6).

(2) ***Confirmation of the Knudsen-Dominated Regime***: We introduced a collision-based analysis directly from the NEMD trajectories to quantify the ratio of gas-wall to gas-gas collisions. This confirms Knudsen transport dominates in our simulations (added Text S2).

(3) ***Clarification of Model Simplifications and Advantages***: We further explained how our model simplifies complex disordered porous networks into a channel with internal barriers (added Fig. S1). Additionally, we discussed the advantages of our framework over conventional tortuosity-based models.

Here, the authors developed a generalized theoretical framework to describe Knudsen flow in disordered porous materials. Specifically, they developed a relationship between permeability, mean pore size, and porosity. They validated their theory against MC simulations with 5000 random porous networks. Furthermore, they performed NEMD simulations to demonstrate the applicability of their theory to realistic membranes such as polyamide. Deriving a relationship for permeability in random porous materials is almost impossible due to the complex channel geometry. However, the authors present a neat solution by simplifying the channels as cylindrical channels with internal barriers, allowing permeability (or transmission probability) to be expressed analytically. This is an elegant engineering approach, and given that the authors validated their theoretical framework, I recommend the manuscript for publication.

Response: We thank the reviewer for the positive comments and recommendation for publication.

I have the following comments and suggestions to further improve the manuscript:

-Currently, transport through the nanoporous material is modeled as a series of barriers in a 1D channel. What happens if a barrier is infinitely high (e.g., representing a dead end inside the porous materials)? Should the model instead be constructed based on multiple channels in parallel?

Response: We thank the Reviewer for this insightful comment. Our current theoretical framework actually accounts for the presence of dead ends. Our model divides the porous network into consecutive layers and evaluates the pore overlap between them, simplifying the complex network into a 1D channel with barriers representing the transport resistance between adjacent layers. A dead end is simply a pore in one layer that faces solid material in the adjacent layer. In a real porous structure, the barrier constriction ratio follows a distribution ranging from 0 to 1. The ratio equals 1 when the corresponding area in the adjacent layer is entirely porous. It equals 0 when the area

is completely solid, which corresponds to a dead end. For partial overlaps, the value falls between 0 and 1. Our analysis shows that in an isotropic porous network, the statistical average of this distribution equals the pore overlap fraction between two adjacent layers. This overlap fraction is equivalent to the bulk porosity. Therefore, the effective barrier constriction ratio for all barriers in the simplified model can be treated as equal to the porosity.

The equivalent 1D barrier-channel model should therefore be viewed as a coarse-grained statistical representation of the ensemble-average transport behavior of many interconnected pathways within the porous network, rather than a literal single transport channel.

- It is not clear how structural features such as pore connectivity are captured in this formulation. For example, connectivity can strongly influence percolation pathways and the presence of dead-end pores. The authors may want to comment on how connectivity is implicitly reflected in parameters such as porosity or barriers.

Response: We thank the reviewer for this insightful comment. We agree that pore connectivity can strongly affect percolation pathways and dead-end pores. In this study, the porous network is divided into consecutive layers, and gas transport is statistically analyzed as transmission between adjacent layers. This treatment allows us to avoid explicitly defining parameters from the perspective of gas pathways, such as connectivity and tortuosity, in a complex 3D network. Omitting these parameters makes it easier to derive concise formulas.

Although connectivity is not included as an independent parameter, its effect is implicitly reflected by porosity and mean pore size. These two parameters determine the pore number density in a random porous network. At a given mean pore size, a higher porosity corresponds to more pores and increases the opportunity for a pore to connect to others, enhancing connectivity. In contrast, a low porosity indicates a low pore number density, which reduces the probability of pore connection and increases the chance of isolated or dead-end pores. Thus, the effect of connectivity is captured through the statistical relation between porosity and mean pore size.

The present framework should thus be viewed as a coarse-grained statistical description of transport in random porous networks, where connectivity effects are incorporated implicitly through averaged structural descriptors rather than explicitly resolved pathway topology.

In the revised manuscript, we added discussion about how connectivity and dead-end pores are implicitly reflected in our model.

Line 433: *"In porous networks, structural features such as pore connectivity and the presence of dead-end pores significantly influence gas transport. Explicitly parameterizing these pathway-dependent properties substantially complicates porous gas flow models. Our equivalent strategy, which divides the network into consecutive stacked layers and statistically evaluates transmission between adjacent layers, avoids the need to explicitly define these complex variables. Nevertheless, the physical effects of connectivity and dead ends are implicitly captured by the system's porosity and mean pore size. In a random isotropic network, these two variables collectively determine the pore number density. At a given mean pore size, a higher porosity corresponds to a greater number of pores and a higher likelihood of pore interconnection and accessible transport pathways,*

whereas lower porosity increases the probability of isolated or dead-end pore regions. Thus, the effect of connectivity and dead-end pores is implicitly incorporated through the ensemble-average transport resistance represented by porosity and mean pore size."

Point-by-point response to the reviewers' comments

Reviewer #1 (Remarks to the Author):

The authors have largely addressed this reviewer's comments appropriately. Nevertheless, the usefulness of the theory remains tenuous. This is because it is applicable to only weakly adsorbing gases, and at normal conditions only helium and for sufficiently large pore sizes, hydrogen, will satisfy this requirement. For most important gases such as methane or carbon dioxide unrealistically high temperatures will be required before the theory becomes applicable.

Response: We thank the reviewer for this comment. Gas transport in porous networks is a complex problem in which viscous flow, Knudsen diffusion, and surface diffusion can coexist, and the structural diversity of porous materials adds further complexity. **We emphasize that <u>we did not intend to provide a universal solution</u> that encompasses all these mechanisms within a single framework**.

The focus of this work is specifically on Knudsen diffusion, <u>which excludes strongly adsorbing gases</u> such as carbon dioxide under ambient conditions. Nevertheless, Knudsen diffusion remains highly relevant for many important applications, including helium and hydrogen membrane transport, high-temperature gas membrane transport, and water vapor transport in hydrophobic materials. We believe that establishing a rigorous and accessible theoretical framework for the Knudsen regime is an important step toward a more complete description of gas transport in disordered porous materials.

In the revised manuscript, we have clearly stated the scope and applicability of the theory.

Line 442: "*First, surface diffusion and capillary condensation can contribute substantially to gas transport in nanoporous materials, particularly for strongly adsorbing gases. As the present theory focuses solely on Knudsen diffusion, it does not capture these mechanisms. Consequently, the framework is applicable to weakly adsorbing gases such as helium and hydrogen, as well as to gas transport carried out at elevated temperatures where adsorption effects are suppressed.*"

A further weakness is that any disordered material will have a pore size distribution, which requires three parameters to describe it. These are the porosity, modal pore size, and standard deviation. The authors characterize a random medium by only the porosity and a single pore radius. How is this able to capture materials with a wide pore size distribution, and the effect of width of the pore size distribution on the permeability?

Response: We thank the reviewer for raising this point. The mean pore size used in our model is calculated by integrating over the pore size distribution (PSD):

$$\bar{d} = \int d \cdot \mathrm{PSD}(d)\mathrm{d}d$$

In Knudsen theory, gas flux scales approximately linearly with pore diameter. Consequently, the first-order effect of the pore size distribution enters through its first moment, namely the mean pore size. When pores of different sizes contribute additively to the total flux, as assumed in our layer-based model, the average flux through a distribution of pores is well approximated by the

flux computed using the mean pore size alone. The mean pore size therefore serves as a physically motivated single parameter that captures the dominant influence of the PSD to gas permeability.

To assess how broadly this applies, we computed the coefficient of variation (CV) of the PSD across the 5,000 disordered structures used in this study. The CV is defined as the ratio of the standard deviation to the mean of the pore size distribution, and serves as a normalized measure of pore size dispersity. As shown in Fig. R1, the CV values of our structural dataset range from approximately 0.4 to 1.0, indicating that the porous structures studied here exhibit a wide variety of pore size distributions. **Despite this broad range of PSD widths, the proposed model achieves excellent agreement with Monte Carlo simulations (R^2=0.986).** This suggests that the mean pore size alone captures the dominant dependence of gas permeability on pore size for the random porous structures considered here.

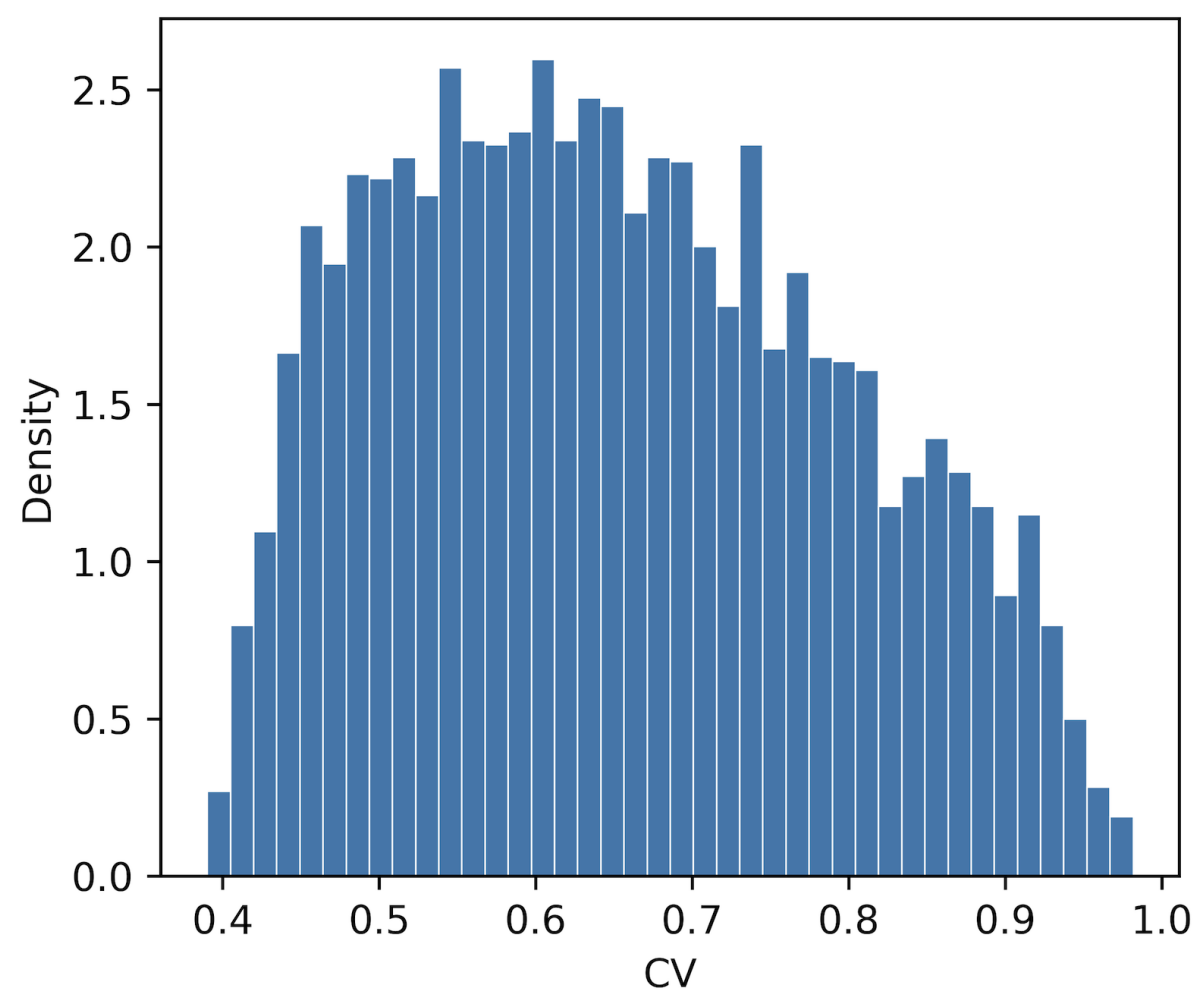

Figure R1. Distribution of the coefficient of variation (CV) of the pore size distribution across 5,000 disordered porous structures. The CV, defined as the ratio of the standard deviation to the mean of the pore size distribution, ranges from approximately 0.4 to 1.0, showing that the porous structures studied here cover a wide range of pore size distributions.

We acknowledge that this remains a simplification. Incorporating additional PSD characteristics such as the distribution width may become important for highly heterogeneous porous materials and could further improve the accuracy of future models. We have clarified this point in the revised manuscript.

Line 456: "*Fifth, our model characterizes the pore size distribution using only the mean pore size. Although the mean pore size is obtained by integrating over the full pore size distribution, other distributional features such as the distribution width are not explicitly accounted for.*"

While I am not recommending that the manuscript be declined, I suggest the authors address these questions and build a stronger case for the importance and use of the approach. Otherwise, I suspect it is unlikely to achieve its intended impact.

Response: We thank the reviewer for the constructive feedback. We have carefully addressed all the raised concerns and revised the manuscript accordingly. We hope the revised version more

clearly demonstrates both the importance of the Knudsen transport regime and the value of the proposed framework within its intended scope.
